# iPSC‐derived NK cells with site‐specific integration of CAR19 and IL24 at the multi‐copy rDNA locus enhanced antitumor activity and proliferation

**DOI:** 10.1002/mco2.553

**Published:** 2024-05-09

**Authors:** Yuxuan Zhang, Qingxin Shi, Peiyun Wang, Chujun Huang, Shuqing Tang, Miaojin Zhou, Qian Hu, Lingqian Wu, Desheng Liang

**Affiliations:** ^1^ Center for Medical Genetics & Hunan Key Laboratory of Medical Genetics School of Life Sciences Central South University Changsha China; ^2^ Hunan Key Laboratory of Animal Models for Human Diseases School of Life Sciences Central South University Changsha China

**Keywords:** chimeric antigen receptors, immunotherapy, induced pluripotent stem cells, interleukin 24, natural killer cells

## Abstract

The generation of chimeric antigen receptor‐modified natural killer (CAR‐NK) cells using induced pluripotent stem cells (iPSCs) has emerged as one of the paradigms for manufacturing off‐the‐shelf universal immunotherapy. However, there are still some challenges in enhancing the potency, safety, and multiple actions of CAR‐NK cells. Here, iPSCs were site‐specifically integrated at the ribosomal DNA (rDNA) locus with interleukin 24 (IL24) and CD19‐specific chimeric antigen receptor (CAR19), and successfully differentiated into iPSC‐derived NK (iNK) cells, followed by expansion using magnetic beads in vitro. Compared with the CAR19‐iNK cells, IL24 armored CAR19‐iNK (CAR19‐IL24‐iNK) cells showed higher cytotoxic capacity and amplification ability in vitro and inhibited tumor progression more effectively with better survival in a B‐cell acute lymphoblastic leukaemia (B‐ALL) (Nalm‐6 (Luc1))‐bearing mouse model. Interestingly, RNA‐sequencing analysis showed that IL24 may enhance iNK cell function through nuclear factor kappa B (NFκB) pathway‐related genes while exerting a direct effect on tumor cells. This study proved the feasibility and potential of combining IL24 with CAR‐iNK cell therapy, suggesting a novel and promising off‐the‐shelf immunotherapy strategy.

## Graphical abstract

Zhang et al. successfully regenerated iNK cells from human iPSCs with rDNA locus gene editing. IL24 enhances the antitumor activity and proliferation of armored CAR‐iNK cells, which may be involved in cellular‐positive upregulation and adhesion pathways.

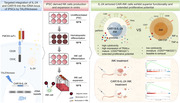

## 1 INTRODUCTION

Currently, a substantial number of studies have indicated that NK cells, especially chimeric antigen receptor‐modified natural killer (CAR‐NK) cells, emerge as an essential player in the field of immunotherapy due to their promising therapeutic efficacy, inherent safety and universal applicability to malignancies.^1^, ^2^ However, several limitations prevent NK cells from being used more broadly. Although better gene transfer efficiency has been achieved via NK cell lines, particularly with viral vectors, manipulation of primary NK cells remains challenging.^3^, ^4^ Moreover, the short post‐infusion persistence of NK cells limits their clinical efficacy.^5^, ^6^ In order to overcome these problems, genetic modifications are made to CAR‐NK cells to improve their persistence, enhance their homing ability, or evade functional and metabolic tumor microenvironment (TME) suppression, which can strengthen the targeting of solid tumors.^1^, ^7^ Cytokines fundamentally contribute to modulating and improving CAR‐NK cell functions. Mainly expressed in immune cells, interleukin 24 (IL24) exerts extensive tumor‐specific cytotoxic effects on a variety of malignancies without affecting normal cells. Evidence suggests that IL24 (also known as MDA‐7) may represent a promising cancer therapeutic agent in further studies,^8^, ^9^, ^10^ while research on IL24 delivery with multiple routes is undergoing continued clinical development (INGN‐241). However, there has not yet been a comprehensive exploration of the efficacy of combining IL24 and CAR‐NK cells.

As a revolutionary technology in regenerative medicine, induced pluripotent stem cells (iPSCs) are considered to be an optimal cellular resource for generating CAR‐NK cells and have the potential to overcome various obstacles encountered by CAR‐NK cells, including cell quantity, quality control, and development of universal off‐the‐shelf products.^11^, ^12^ The unlimited reproduction ability of iPSCs enables the manipulation of genetic modifications without the concern of cell exhaustion, even when working with cell products derived from mature fully differentiated immune cells.^13^ However, it is required that the execution of various engineering procedures should enhance the therapeutic efficacy of iPSC‐derived NK (iNK) cells, which remains a substantial problem that should be addressed thoroughly.

Studies have shown that the ribosomal DNA (rDNA) region is characterized by multi‐copy repetition and is considered a safe and excellent location for integrating exogenous genes.^14^, ^15^ By integrating and expressing multiple copies at the rDNA locus, stem cell‐based gene therapy strategies have been previously established in our laboratory, which achieved antitumor effects in vivo and in vitro.^16^, ^17^

This study provided a novel treatment for CAR‐NK cells in which IL24 is integrated into the rDNA locus that followed an improved differentiation and amplification protocol, and combined strategies suggest the potential for greater therapeutic effectiveness.

## 2 RESULTS

### 2.1 Varying concentrations of rIL24 failed to inhibit both NK cells and iNK cells

To clarify the effects of IL24 on NK cells, the viability and proliferation of both peripheral blood (PB) NK cells and peripheral blood mononuclear cells (PBMCs) treated with varying concentrations (0.2‒200 ng/mL) of recombinant IL24 protein (rIL24) were first tested. To be specific, the viability of isolated and purified CD56^+^ NK cells was enhanced by a broad range of rIL24 treatments (0.2‒200 ng/mL), in which rIL2 (200 IU/mL) treatment was used as a positive control (Figure S1A). In long‐term culture (10 days), there was no significant influence of rIL24 on the proliferation, the proportion of CD56^bright^CD16^−^ and CD56^dim^CD16^+^ subsets in NK cells, and cell cycle (Figure S1B‒D). Similarly, PBMCs from healthy adults were cultured with rIL24 only and no significant differences were observed in comparison with the blank group (Figure S1E‒G). Next, the impact of rIL24 on the differentiation of iPSCs into NK cells by measuring NK cell maker (CD56) was analyzed. After 38 days of culture with an additional variant concentration of rIL24, both the treated and untreated groups demonstrated similar efficiency in differentiation (Figure S1H). Moreover, no statistical significance on the expression of surface markers related to early activation (CD69) and exhaustion (PD‐1, Tim‐3, and LAG‐3) was observed, indicating that the activation and functionality of NK cells were not impaired during differentiation in vitro (Figure S1I,J). All of these results indicate the feasibility of armoring IL24 to CAR19‐NK cells.

### 2.2 Targeted integration of *IL24* and *CAR19* into the rDNA locus of iPSCs by TALENickases‐mediated homologous recombination

**FIGURE 1:**
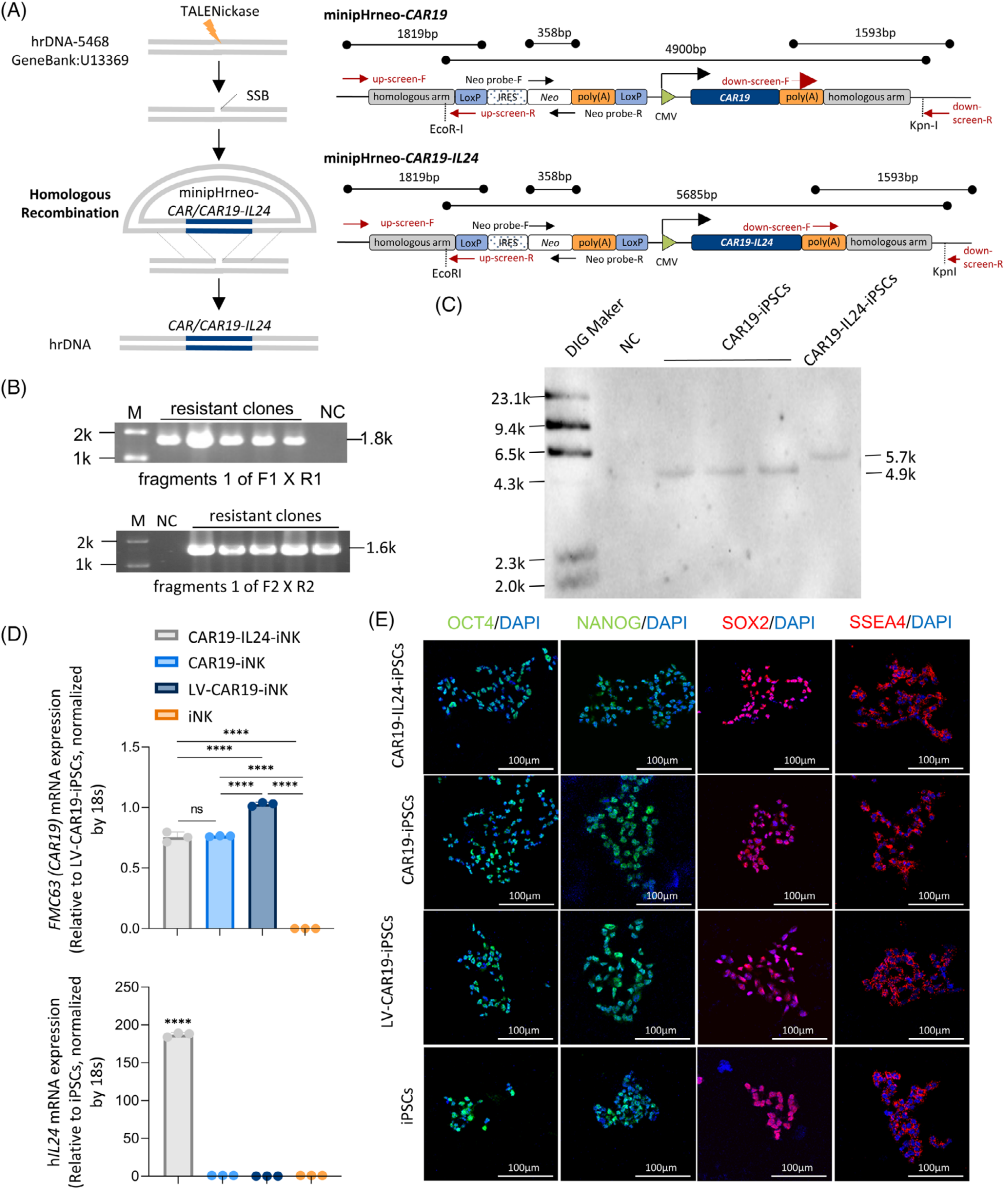
Generation of CAR19‐IL24‐iPSCs by TALENickases‐mediated ribosomal DNA (rDNA) site‐specific integration. (A) Flow chart of rDNA targeting minipHrneo‐*CAR*/minipHrneo‐*CAR‐IL24* (blue segments). Gray boxes indicate the 1593‐bp right homologous arm (RHA) (GenBank U13369: 5468−6064) and 1819‐bp left homologous arm (LHA) (GenBank U13369: 4533−5467). Binding sites of down‐screen‐F/R and up‐screen‐F/R primers are presented. The use of neo‐screen‐F/R to identify neomycin removal binding outside the deleted region. (B) Polymerase chain reaction (PCR) screening of homologous recombination. G418‐resistant induced pluripotent stem cell (iPSC) clones were selected for verification by PCR with down‐screen‐F/R and up‐screen‐F/R, and if homologous recombination were to take place in cells, it would generate 1593 and 1819‐bp PCR products, respectively. (C) Southern blotting analysis demonstrated specific integration. Clones were confirmed by Southern blotting with a 358‐bp DIG probe in the Neo cassette for homologous recombination at the rDNA locus. The arrows indicate the expected 4.9k and 5.7k restriction fragments digested by EcoRI and KpnI. DNA Molecular Weight Marker II was used for the DIG marker. (D) Relative mRNA expression of *CAR19*/h*IL24* in CAR‐iPSCs and unmodified iPSCs. The data represent findings from three independent experiments. (E) Immunofluorescence of the iPSC markers OCT4, NANOG, SOX2, and SSEA‐4. The transformed iPSC clones showed a normal expression and localization consistent with that of normal iPSCs. 4′,6′‐Diamidino‐2‐phenylindole (DAPI) was employed to stain the nuclei, with a scale bar indicating 100 µm. Each group comprised *n* = 5 samples. GraphPad Prism8 is used to analyze all data with error bars, which are expressed as mean ± standard deviation (SD). CAR19, CD19‐specific chimeric antigen receptor; IL24, interleukin 24; M, marker; NC, negative control.

Before gene editing, the human iPSC (hiPSC) lines established previously by our laboratory from the PB T cells of healthy volunteers were employed by using an episomal reprogramming approach (Figure S2A). In past investigations, our laboratory has built minipHrneo, which was a non‐viral vector designed to target human rDNA locus, involving the non‐promoter neomycin (Neo) cassette paired with loxP sequences at both ends.^16^, ^17^ As a proof of concept, we designed the donor vector containing classic CD19‐specific CAR gene (*CAR19*, consisting of FMC63scFv‐CD28TM‐28z) or a combination of *IL24* and *CAR19* (*CAR19‐IL24*) (Figure 1A). In addition, a lentivirus‐mediated randomized integration CAR19‐iPSCs (LV‐CAR19‐iPSCs) was also generated as a control (Figure S2B).

To test the potential of the rDNA region in the engineering of iPSC‐iNK cells, nucleofection was used to guide the minipHrneo plasmid and the donor vector into the rDNA 5468 site of iPSCs (Figure 1A). Following G418 selection, single clone clumps were picked manually and screened by polymerase chain reaction (PCR) (Figures 1B and S2C). Five out of 14 clones (35.7%) for CAR19‐iPSCs and three out of 10 clones (30%) for CAR19‐IL24‐iPSCs were recognized as positive clones by Sanger sequencing, which was consistent with the theoretical sequences (Figure S2D). These positive clones were further validated using Southern blotting and a single 5.7 and 4.9 kb restriction fragment was detected in CAR19‐IL24‐iPSCs and CAR19‐iPSCs, respectively (Figure 1C). At the same time, no abnormality was observed at the three top‐rank predicted off‐target sites (Figure S2E). The above results suggested that both *CAR19* and *CAR19‐IL24* could be precisely integrated at the rDNA 5468 site of iPSCs and that there were no mutations near targeted sequences.

Compared with CAR19‐iPSCs, CAR19‐IL24‐iPSCs showed similar expression of *CAR19* (*FMC63*), but expressed a higher (∼180‐fold) level of h*IL24*. LV‐CAR19‐iPSCs expressed the greatest level of *CAR19* (Figure 1D). According to immunofluorescence analysis and karyotyping, all integrated iPSCs expressed typical human pluripotent stem cell markers (OCT4/SOX2/NANOG/SSEA‐4) and presented normal karyotypes (Figures 1E and S2F). Besides, all engineered iPSCs formed teratomas with all three germ layers after being injected subcutaneously into NOD.Cg‐*Prkdc* ^*scid*^ *Il2rg^em1Smoc^* (NSG) mice (Figure S2G). These results showed that the integration of CAR19 or CAR19‐IL24 in the rDNA region could be expressed robustly without affecting the characteristics of iPSCs.

### 2.3 Generation and characterization of CAR‐loaded iNK cells

**FIGURE 2:**
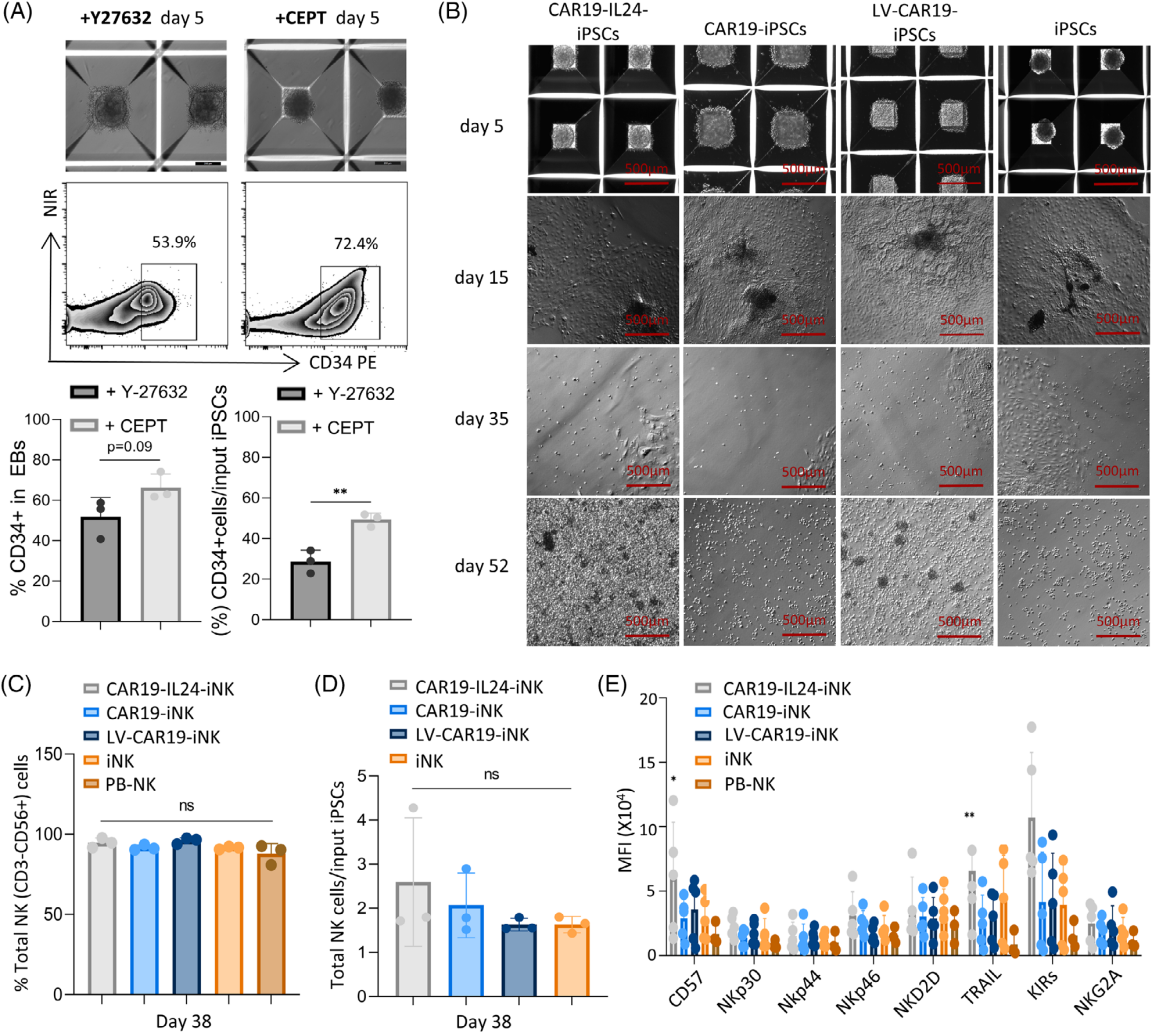
Generation and expansion of CAR‐iNK cells from genetically modified induced pluripotent stem cells (iPSCs). (A) Comparison of embryoid body (EB) formation and yield using different small molecules. The application of CEPT (the mixture of Chroman 1, Emricasan, Polyamines, and Trans‐ISRIB) was favorable for EB formation and could contribute to higher CD34^+^ frequency and yield. The data represent findings from three independent experiments. (B) Morphological changes during natural killer (NK) differentiation and expansion in vitro (scale bar = 500 µm). (C) Identification of iPSC‐derived NK cells on day 38. Statistical results showed the differentiation frequency of total NK cells (CD56^+^) detected by flow cytometry with CD3 gated. Peripheral blood NK (PB‐NK) cells were used as a control. The data represent findings from three independent experiments. (D) Histogram showing the frequency of iPSC‐derived NK differentiation. At the end of day 38 of differentiation, CD3^−^CD56^+^ cells (representing NK cells) are harvested and calculated relative to iPSCs on day 0. The data represent findings from three independent experiments. (E) The statistical findings from flow cytometric analysis of NK cell surface receptors within the CD56+ NK cell populations' gate are shown. These data are reflective of a minimum of five independent experiments. GraphPad Prism8 is used to analyze all data with error bars, which are expressed as mean ± standard deviation (SD). (A) Unpaired two‐tailed Student's *t*‐test; (C and D) one‐way analysis of variance (ANOVA) with Bonferroni correction; (E) two‐way ANOVA with Dunnett correction. ns, not significant, *p* > 0.05; ^*^ *p* < 0.05, ^**^ *p* < 0.01, ^***^ *p* < 0.001, ^****^ *p* < 0.0001. CAR, chimeric antigen receptor.

To produce iNK cells, an embryoid body (EB)‐based two‐stage differentiation procedure was conducted (Figure S3A). It has been found that hiPSCs and hESCs are differentiated into NK cells that exhibit comparable characteristics and functions to NK cells obtained from PBMCs.^18^, ^19^, ^20^ By applying the CEPT cocktail (the mixture of Chroman 1, Emricasan, Polyamines, and Trans‐ISRIB) at day 0,^21^ CEPT enhanced EB formation to reduce the loss of input iPSCs (including epithelial cell‐derived iPSCs) and promoted the generation of CD34^+^ hematopoietic cells, which improved the differentiation efficiency (Figures 2A and S3B‒D). All types of iNK cells generated from iPSCs exhibited the same morphological alterations during differentiation in vitro (Figure 2B).

Then, we evaluated NK cell surface receptors within the gate of CD45^+^CD3^−^CD56^+^ NK cell populations, including NK lineage marker CD56/16 and functional markers (CD57/Nkp30/Nkp44/Nkp46/NKG2D/TRAIL/KIRs/NKG2A) (Figure S3E). When comparing the yield of total NK cells (CD3‒CD56+) and total NK cells/input iPSCs, these CAR‐expressing iPSCs showed comparable efficacy in NK cell production to iPSCs (Figure 2C,D and S3F). Moreover, the phenotype of four iNK cells also had similarities to PB‐NK cells (Figures 2E and S3G). Notably, CD57 (correlates with NK cell maturation and high cytotoxic potential) and TRAIL expressions were more significantly elevated compared to those of the other three (Figure 2E), which suggested that CAR19‐IL24‐iNK cells may have enhanced antitumor potential.^22^

**FIGURE 3:**
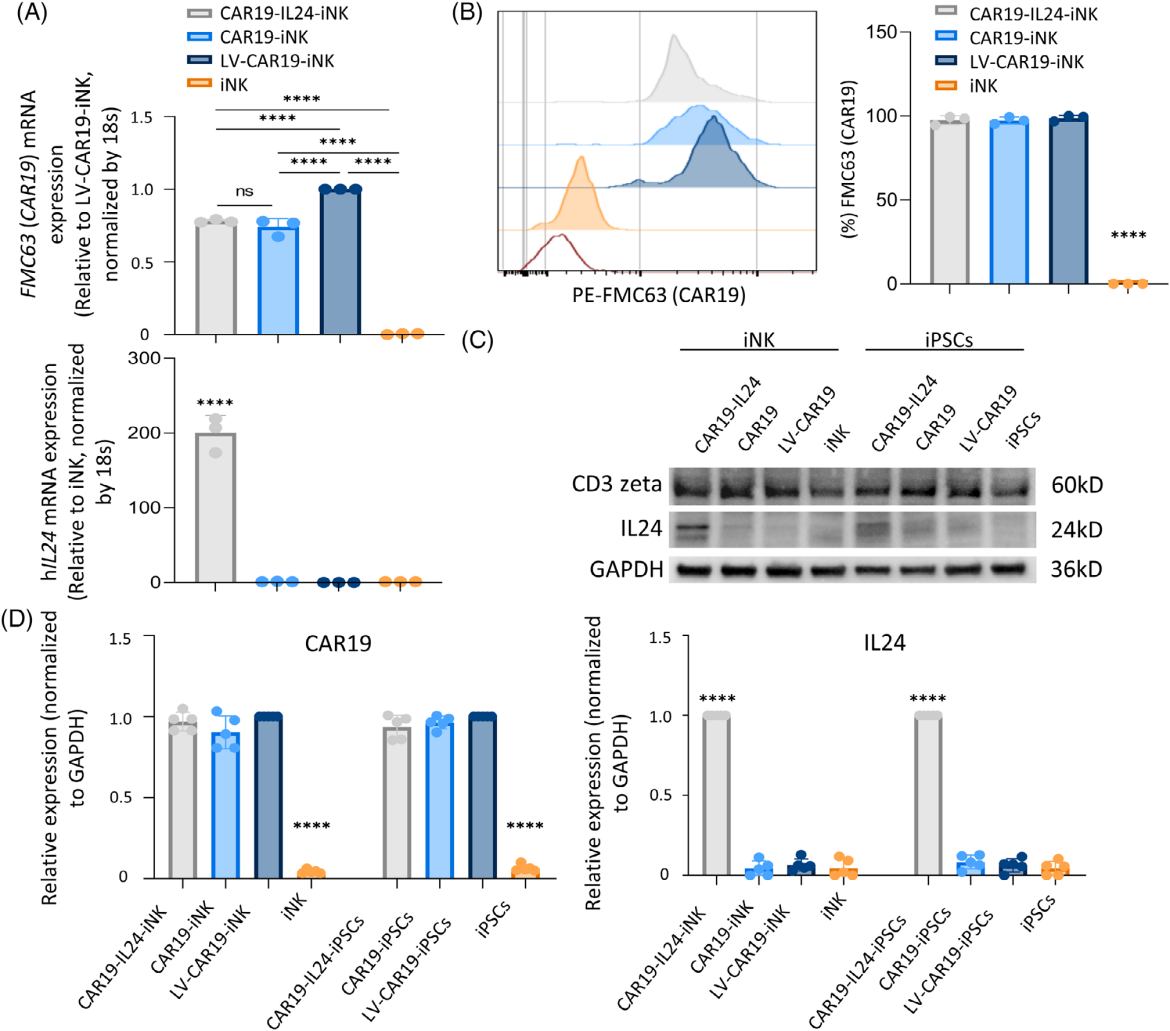
Expression of CD19‐specific chimeric antigen receptor (CAR19) and hIL24 in CAR‐loaded iPSC/iNK cells. (A) The relative mRNA expression levels of *CAR19*/h*IL24* in CAR‐iNK cells and unmodified iNK cells. The data presented are representative of a minimum of three independent experiments. (B) Expression of FMC63 (representing CAR19) in CAR‐iNK cells. Representative flow cytometry data (left) and statistical results (right) showed the FMC63 surface expression of total natural killer (NK) cells. The red line refers to the isotype control. The data presented are representative of a minimum of four independent experiments. (C) Immunoblot analysis of CAR19 and hIL24 protein in the cell lysate of CAR‐iNK/iPSC cells. Left: CAR19; right: hIL24. The expression of CAR was determined using an anti‐CD3ζ antibody, with the molecular size estimated. GAPDH served as the loading control. (D) Quantification of the total CAR19 (left) and hIL24 (right) protein levels in CAR‐iNK/iPSC cells. The data are representative of five independent experiments. GraphPad Prism8 is used to analyze all data with error bars, which are expressed as the mean ± standard deviation (SD). (A) One‐way analysis of variance (ANOVA) with Bonferroni correction (top) and two‐way ANOVA with Dunnett correction (below). (B and D) Two‐way ANOVA with Dunnett correction. ns, not significant, *p* > 0.05; ^*^ *p* < 0.05, ^**^ *p* < 0.01, ^***^ *p* < 0.001, ^****^ *p* < 0.0001. IL24, interleukin 24; iPSC, induced pluripotent stem cell.

The expression of *CAR* and *IL24* in four iNK cells was next analyzed. At the endpoint of the differentiation protocol (day 38), quantitative PCR was adopted to initially evaluate the expression of *CAR19* and h*IL24* (Figure 3A). The four iNK cells had similar expression levels, consistent with those of the iPSCs stage. Also, the protein expression of iNK cells was validated by flow cytometry and immunoblots. CAR19‐IL24‐iNK, CAR19‐iNK, and LV‐CAR19‐iNK cells exhibited similar expression levels, and the expression of CAR‐loaded iPSCs was determined based on FMC63 (representing CAR19) expression (flow cytometry) and detection of CD3 zeta (immunoblots) in cell lysates, while a greater concentration of IL24 was observed in cell lysis of CAR19‐IL24‐iNK cells (Figure 3B‒D). Collectively, iPSCs integrated with *CAR19* and *IL24* at the rDNA locus can be successfully differentiated into NK cells.

### 2.4 Expansion of iPSC‐derived NK cells based on magnetic beads method in vitro

**FIGURE 4:**
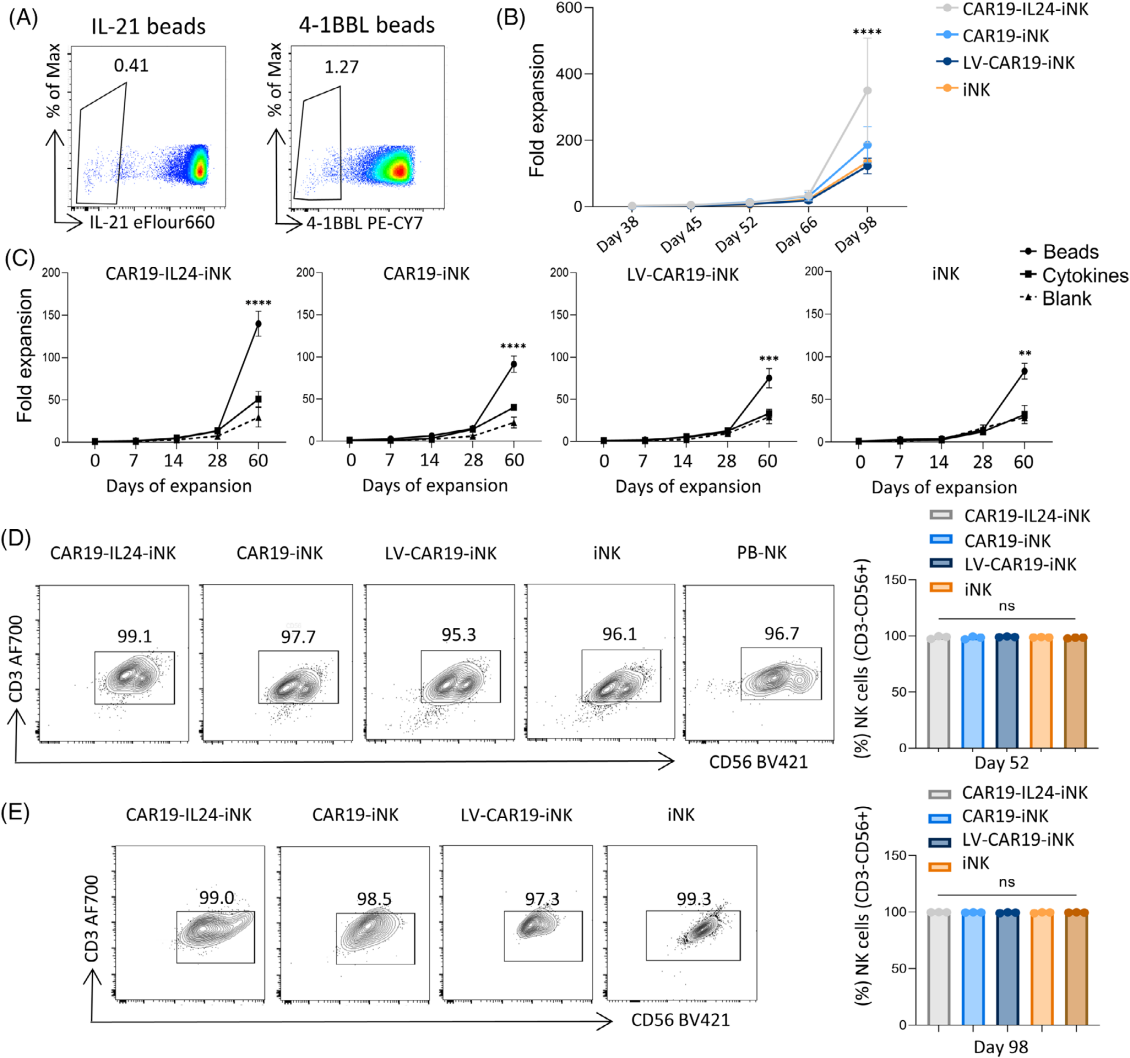
Expansion of CAR‐iNK cells by using IL21/4‐1BBL magnetic beads. (A) Flow cytometry data representative of the coupling results of IL21/4‐1BBL magnetic beads are presented. (B) Summary of induced pluripotent stem cell (iPSC)‐derived natural killer (NK) cells using beads during the expansion procedures. CD3^−^CD56^+^ cells (representing NK cells) were harvested at different checkpoints and data were calculated relative to day 0. The data represent findings from three independent experiments. (C) Expansion kinetics of iPSC‐derived NK cells using beads and cytokines. Data were calculated relative to day 0. The data represent findings from three independent experiments. (D and E) Identification of iPSC‐derived NK cells (CD3^−^CD56^+^) after 14 days (day 52, D) and 60 days (day 98, E) of expansion. Representative flow cytometry data (left) and statistical results (right) showed the proportion of total NK cells. Peripheral blood (PB) NK cells were used as a control on day 52. The data presented are representative of three independent experiments. GraphPad Prism8 is used to analyze all data with error bars, which are expressed as mean ± standard deviation (SD). (B and C) Two‐way analysis of variance (ANOVA) with Dunnett correction; (D and E) one‐way ANOVA with Bonferroni correction. ns, not significant, *p* > 0.05; ^*^ *p* < 0.05, ^**^ *p* < 0.01, ^***^ *p* < 0.001, ^****^ *p* < 0.0001. CAR, chimeric antigen receptor; IL24, interleukin 24.

Due to safety and purity concerns, a feeder‐free, bead‐based amplification technique was employed to risk off exogenic cells in this study (Figure S3H). The use of immobilized human IL21 and 4‐1BBL resulted in notable expansion of NK cells with exceptional purity and superb cytotoxicity from PBMCs previously.^23^ Following stimulation and expansion of 60 days, all of those four groups shared a similar expanding curve following 60 days of expansion, while CAR19‐IL24‐iNK cells exhibited a more dramatic growth capacity (Figure 4A,B). Of note, our data revealed that the bead amplification technique produced much larger proliferation than the cytokines addition technique in all iNK cells (Figure 4C). Additionally, the purity of CD3^−^CD56^+^ NK cells remained above 95% with no extra enrichment during the expansion course (Figure 4D,E). Although there is certainly room for improvement, future applications of bead expansion remain promising.

### 2.5 Functionality of CAR‐loaded iPSC‐derived NK cells responds to CD19^+^ targets

**FIGURE 5:**
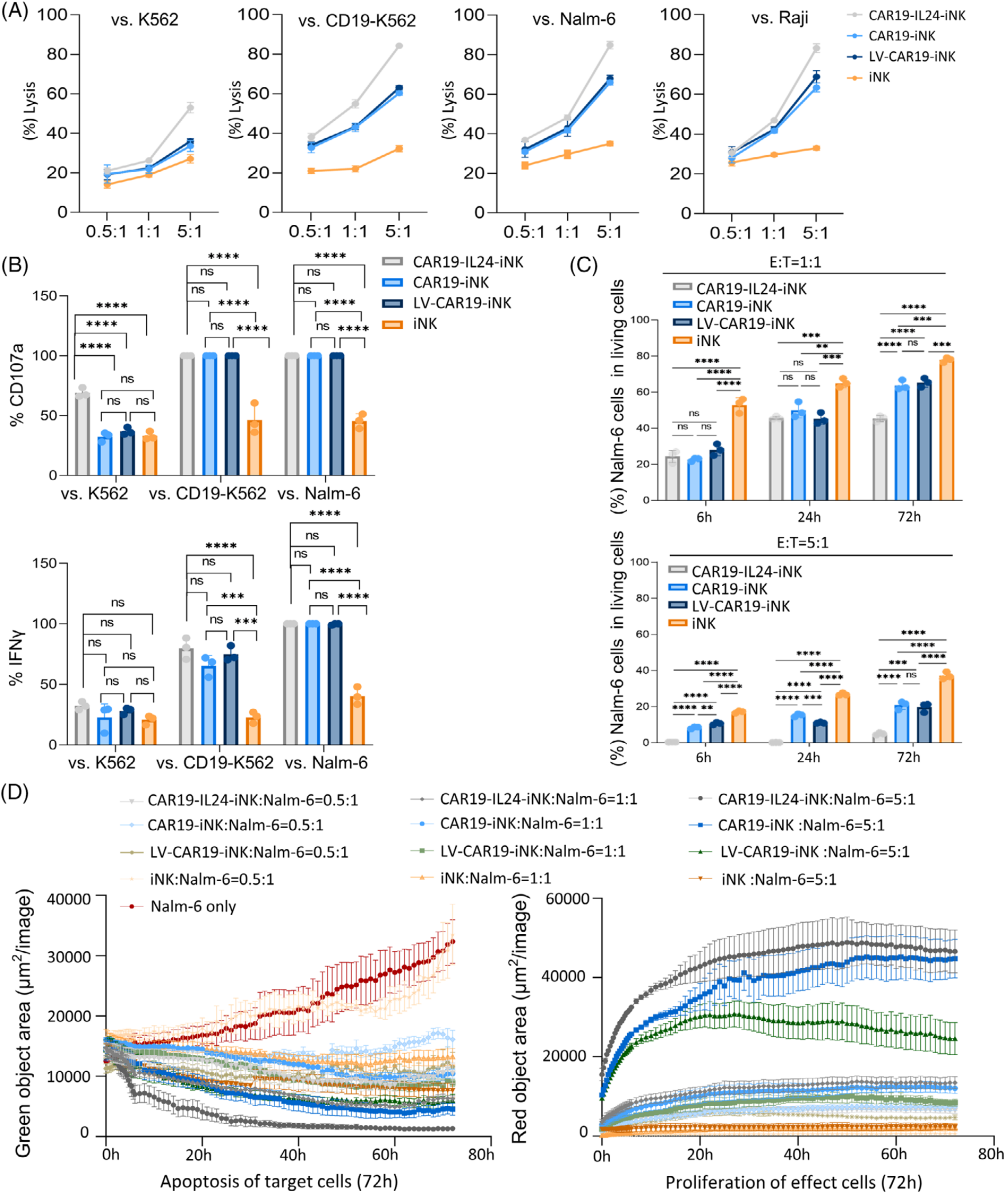
Cytotoxicity and proliferation of anti‐CD19 CAR‐iNK cells against cancer cells in vitro. (A) Quantification and statistics of the lactate dehydrogenase (LDH) release in K562, CD19‐K562, Nalm‐6, and Raji cell lysis. The data represent findings from three independent experiments. (B) Release of CD107a and interferon‐gamma (IFN‐γ) after being challenged with K562 (CD19^‒^), CD19‐K562 (CD19^+^), and Nalm‐6 (CD19^+^) cells. Quantification and statistics of flow cytometry analysis of CD107a (top) and IFN‐γ (bottom) are shown. The data represent findings from three independent experiments. (C) Quantitative and statistical results showed the apoptosis in far red‐labeled Nalm‐6 cells at different E:T ratios. The data represent findings from three independent experiments. (D) Cytotoxic of CAR‐iNK cell population against Nalm‐6 cells was quantified using the IncuCyte real‐time imaging system over a 72‐h time course. Target cells (green‐labeled Nalm‐6) co‐cultured with effect cells (red‐labeled iNK) were monitored. Apoptosis of Nalm‐6 cells (left) and proliferation of iNK cells (right) were measured by counting the pre‐labeled nuclei. GraphPad Prism8 is used to analyze all data with error bars, which are expressed as the mean ± standard deviation (SD). (B and C) One‐way analysis of variance (ANOVA) with Bonferroni correction. ns, not significant, *p* > 0.05; ^*^ *p* < 0.05, ^**^ *p* < 0.01, ^***^ *p* < 0.001, ^****^ *p* < 0.0001. CAR, chimeric antigen receptor; NK, natural killer.

The function of differentiated iNK cells was investigated by in vitro co‐culture assays with various lymphoma cells, including K562 (CD19‒, CML), CD19‐K562 (CD19+), Nalm‐6 (CD19+, ALL), and Raji (CD19+, Burkitt's). To assess the activity of the CAR‐loaded iNK cells in vitro, short‐term cytotoxicity was tested by adopting the lactate dehydrogenase release assays. According to expectation, three CAR‐loaded iNK cell groups exhibited better antitumor capacity and more effective killing effects compared with iNK cells in a CD19‐specific and dose‐dependent manner (Figure 5A). Then, the expression of CD107a (granule release) and interferon‐gamma (IFN‐γ) was assayed by flow cytometry via being stimulated by K562, CD19‐K562, and Raji cells, respectively. Compared with other groups, the CAR19‐IL24‐iNK group produced higher levels of CD107a and IFN‐γ, which implied a more cytotoxic response to stimulation (Figures 5B and S4A). Furthermore, the long‐term cytotoxicity of four iNK cells (labeled with Tag‐it Violet dye) was evaluated by co‐culture with tumor cells (labeled with CellTrace Far Red) (Figure S4B). Since there was the same proportion of target cells initially inoculated in the co‐culture system, the CAR19‐IL24‐iNK co‐culture group showed minimum residual tumor cells, which indicated a robust killing effect (Figure 5C).

According to clinical data, IL24 was observed to be downregulated in tumor tissues, and low IL24 expression was determined to be a prognostic indicator of poor outcomes in Burkitt lymphoma patients.^24^ In this work, we noted that IL24 secreted by CAR19‐IL24‐iNK cells (∼131 pg/10^6^ cells/24 h) was five times higher than CAR19‐iNK cells (∼25 pg/10^6^ cells/24 h) using enzyme linked immunosorbent assay (ELISA) (Figure S4C, left). Given the safety concern that elevated levels of systematic IL24 may cause inflammatory risks and even unexpected side effects, type II interferon (IFN‐γ)/ tumor necrosis factor alpha (TNF‐α)/ granulocyte macrophage‐colony stimulating factor (GM‐CSF) in iNK cells supernatant was also evaluated by ELISA. The results indicated that CAR19‐IL24‐iNK cells produced higher levels of TNF‐α but lower levels of IFN‐γ and GM‐CSF, which were related to a reduced risk of cytokine release syndrome (CRS) and neurotoxicity in comparison with CAR19‐iNK cells (Figure S4C). Following the prior reports, a decreased level of systematic IFN‐γ might be safer.^25^ The effect of cell‐mediated apoptosis of target cells (Annexin V in Nalm‐6 cells) decreased in long‐term co‐culture, in which the CAR19‐IL24‐iNK group consistently showed a stronger role in mediating tumor cell apoptosis (Figures 5C and S4D).

As CAR19‐IL24‐iNK cells expand faster than the other iNK cells, we next evaluated the expansion capacity by staining iNK cells. Due to steady proliferation (72 h vs. 0 h) irrespective of tumor stimulus, the CAR19‐IL24‐iNK group presented more decay of staining strength, which suggested a better survival ex vivo (Figure S4E). Furthermore, we compared the antitumor effect of distinct CAR‐loaded iNK cells by using the Incucyte Live‐Cell Analysis System. Here, target cells (green‐labeled Nalm‐6) co‐cultured with effector cells (red‐labeled iNK) at variant E:T ratios (0.5/1/5) were monitored by Incucyte over 72 h. By counting the green‐labeled nuclei, apoptosis of tumor cells was measured. It was found that CAR‐loaded iNK cells reduced green object area (tumor cells) to different degrees, in which CAR19‐IL24‐iNK cells (gray) demonstrated superior antitumor activity particularly at high effector‒target ratios over time. Meanwhile, CAR19‐IL24‐iNK cells (gray) consistently exhibited more robust proliferation (red object area) regardless of the E:T ratio (Figure 5D). These findings suggested that CAR‐loaded iNK cells, especially CAR19‐IL24‐iNK cells, could effectively enhance the killing effect sustain cytotoxicity, and exhibit improved proliferation. This may contribute to better survival in tumor elimination.

### 2.6 CAR19‐IL24‐iNK cells harness the antitumor activity and proliferation in vivo

**FIGURE 6:**
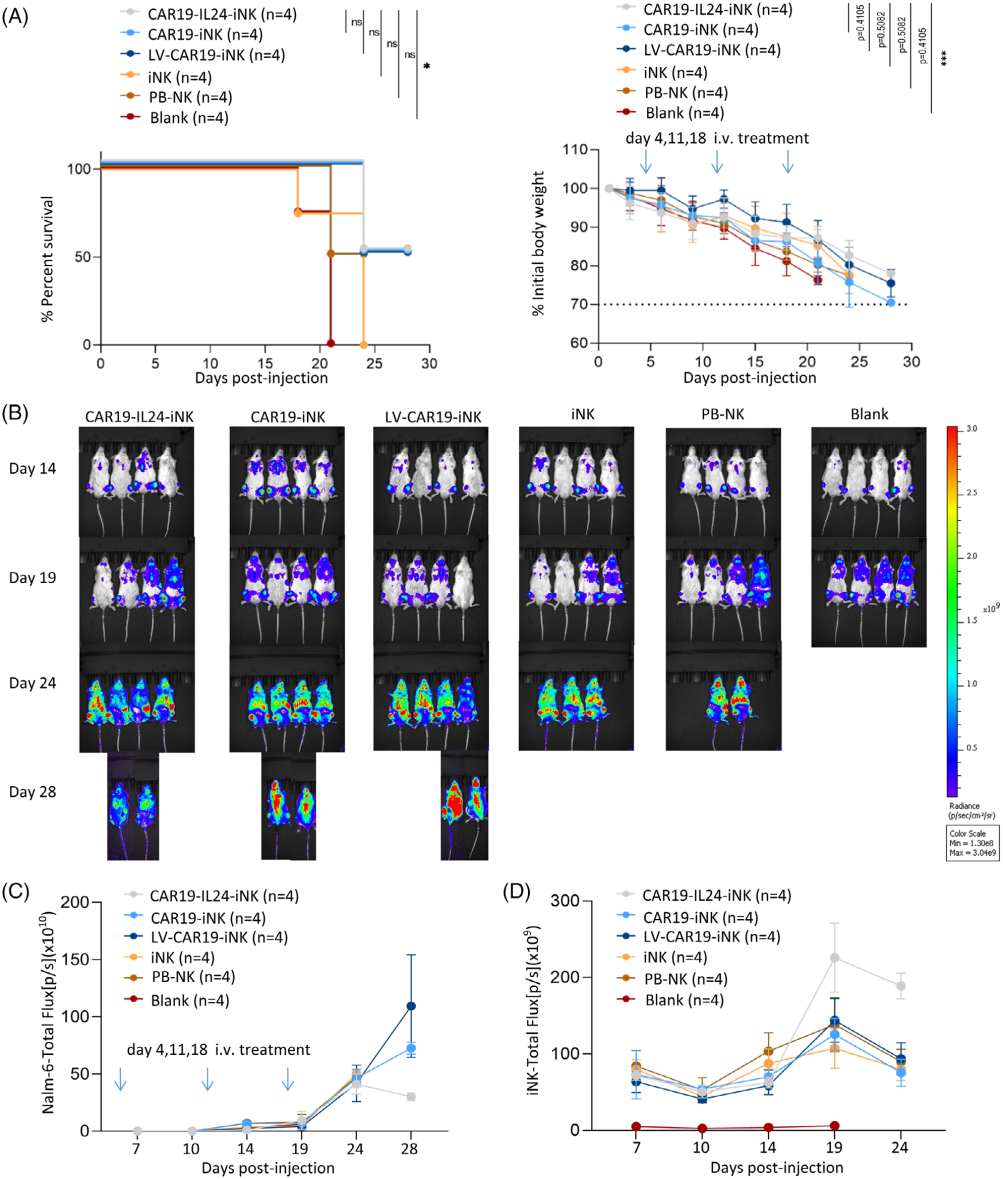
Antitumor activity of anti‐CD19 CAR‐iNK cells in vivo. (A) Summary of the xenograft mice survival and body weight. NSG mice were inoculated intravenously (i.v.) with Nalm‐6 (Luc1) cells (1 × 10^6^). On days 4, 11, and 18, mice were treated with tail‐vein injection of DiR‐labeled induced pluripotent stem cell (iPSC)‐derived natural killer (NK) cells, peripheral blood NK (PB‐NK) cells, and Dulbecco's phosphate buffered saline (DPBS) (blank) (3 × 10^6^). Survival was summarized with four mice in each group (left). Data were shown using the Kaplan‒Meier survival analysis and *p*‐values were calculated using the log‐rank test (two sided). Relative body weight was shown with four mice/groups (right). *p*‐Values were analyzed using two‐way analysis of variance (ANOVA) with repeated measurements and corrected using the Holm‒Sidak method on day 24. Data are presented as mean ± standard deviation (SD). (B) Tumor burden was assessed over time for each animal using bioluminescent imaging (BLI). Tumor growth in the xenograft model was assessed by quantifying alterations in tumor bioluminescence on Nalm‐6 cells (labeled Luc1) on days 7, 10, 14, 19, 24, and 28. (C) Quantification of Nalm‐6 cells in each animal over time (*n* = 4). Tumor growth in the xenograft model was monitored by measuring changes in tumor BLI total flux (photons/s) on days 7, 10, 14, 19, 24, and 28. (D) Quantification of therapeutic cells in each animal over time (*n* = 4). Summary data of therapeutic cells in the xenograft model were monitored by measuring changes in NK cells' BLI total flux (photons/s) on days 7, 10, 14, 19, and 24. GraphPad Prism8 is used to analyze all data with error bars, which are expressed as mean ± standard deviation (SD). CAR, chimeric antigen receptor.

The short‐term efficiency and safety of CAR19‐IL24‐iNK cells in the Nalm‐6 (Luc1) tumor‐bearing mice model were preliminarily evaluated (Figure S4F). In general, groups treated with CAR‐loaded iNK cells showed both minor weight loss and improved survival (Figure 6A). To investigate whether CAR19‐IL24‐iNK cells still strengthened antitumor activity in vivo, the tumor growth was monitored by measuring the fluorescence signal of Nalm‐6 (Luc1) and proliferation of iNK cells labeled with DiR by bioluminescence imaging (BLI) over time. BLI analysis showed that the CAR19‐IL24‐iNK group was the most efficient decrease in tumor burden among all treated groups even though there was no statistical significance (Figure 6B,C and S4G). In addition, it was also observed that CAR19‐IL24‐iNK cells increased significantly in vivo (Figure 6D).

Moreover, the appropriate infusion dose represents another important factor that should be clarified. Given toxicity and other extension issues, determining the optimal dose based on disease burden or injection frequency remains to be explored.^26^ Therefore, it is essential to improve the function and toxicity of CAR19‐IL24‐iNK cells in future trials.

### 2.7 CAR‐loaded iNK cells armored with IL24 possess promoted cytotoxicity and activity

**FIGURE 7:**
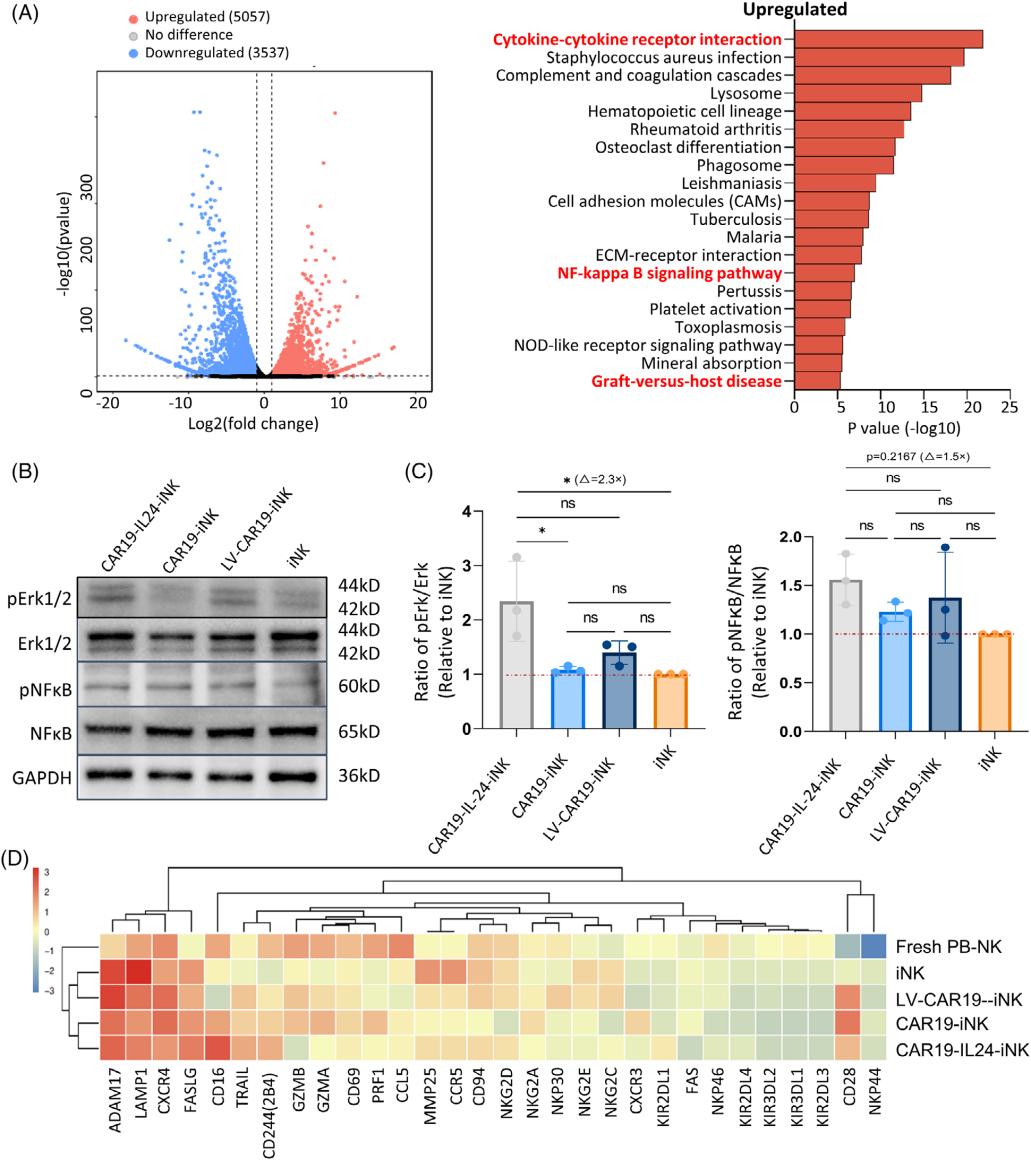
Chimeric antigen receptor (CAR)‐loaded iNK cells armored with interleukin 24 (IL24) possess promoted cytotoxicity and activity. (A) Volcano plot depicting dysregulated genes based on the differential expression data between CAR19‐IL24‐iNK cells and CAR19‐iNK cells (left). Significantly upregulated genes are denoted by red dots, downregulated genes by blue dots, while unchanged genes are represented by gray dots. Kyoto Encyclopedia of Genes and Genomes (KEGG) analysis of the results from the left plot is depicted on the right, which shows the top 20 terms enriched in the upregulated gene sets. Terms related to immune responses are highlighted in bold, red font. (B) Immunoblots were employed for the total and phosphate‐protein analysis of nuclear factor kappa B (NFκB) and Erk1/2 in the cell lysate of iNK cells. (C) Measurement of phosphorylated levels relative to the total levels in natural killer (NK) effector cells co‐cultured with target cells was conducted for Erk (left) and NFκB (right) proteins. Data were normalized using the values of endogenous GAPDH. The data represent findings from three independent experiments. (D) Heatmaps showing normalized read count values of the genes that are essential for NK cell cytotoxicity. *n* = 3 for each group. GraphPad Prism8 is used to analyze all data with error bars, which are expressed as mean ± standard deviation (SD). (C) One‐way analysis of variance (ANOVA) with Bonferroni correction. ns, not significant, *p* > 0.05; ^*^ *p* < 0.05. CAR19, CD19‐specific chimeric antigen receptor.

Utilizing CAR‐engineered NK cells in cancer immunotherapy holds great promise, but the molecular mechanisms underlying their antitumor phenotype and the potential impact of IL24 on CAR‐loaded iNK cells remain unclear. To explore the antitumor phenotype of CAR‐loaded iNK cells and the likely role of IL24, RNA‐sequencing was performed to compare the four iNK cells with PB‐NK cells (GSE165498 downloaded from the Gene Expression Omnibus [GEO] database).^27^ The gene expression profiles revealed a high degree of similarity between IL24 armored or not CAR‐loaded iNK cells, while PB‐NK cells exhibited a different gene expression profile (Figure S5A). For further analyzing the molecular mechanism that underlies the antitumor phenotype of CAR19‐IL24‐iNK cells, differential gene expression analysis was conducted, and 8594 dysregulated genes versus CAR19‐iNK cells were identified, which included 5057 upregulated and 3537 downregulated genes (Figure 7A, left). In the Kyoto Encyclopedia of Genes and Genomes (KEGG) analysis, a predominant enrichment of the genes related to immune responses was revealed, including cytokine‒cytokine receptor interaction and nuclear factor kappa B (NFκB) signaling pathway, particularly within the upregulated gene set (Figure 7A, right). To elucidate the cell signaling pathways mediating CAR‐induced cellular activations more comprehensively, gene set enrichment analysis (GSEA) was carried out on the genes that were most notably upregulated in CAR19‐IL24‐iNK cells versus iNK cells. The genes were discovered to be significantly enriched in the biological process directly related to NK cell proliferation or function in addition to neutrophil activation and chemotaxis (Figure S5B,C). Via Western blot, the expression changes of several degranulation and cytokine production‐related NK cell signaling mediators, which involved NFκB, Erk1, and Erk2, were evaluated.^12^ Notably, CAR19‐IL24‐iNK cells demonstrated an increased ratio of phosphorylation in both NFκB (∼1.5‐fold) and Erk1/2 (∼2.3‐fold) compared to iNK cells (Figure 7B,C). Besides, this activation causes an increase in the expansion and survival of the CAR‐loaded iNK cells that enables improved antitumor activity.^12^ Attention was also paid to the transcription of genes crucial for the cytotoxicity of NK cells, such as FAS and TNFSF10 (Figure 7D). Since IL24 activates multiple signaling cascades in immune cells to induce myeloid cell migration,^10^ it is proposed that IL24 can attract neutrophils and upregulate genes responsible for the downstream signaling of FAS and TNFSF10 to enhance the efficacy of cancer immunotherapy (Figure 7A,D), which was consistent with our previous findings of phenotype and function in CAR19‐IL24‐iNK cells. These results demonstrate that CAR‐loaded iNK cells armored with IL24 possess promoted cytotoxicity and activity.

## 3 DISCUSSION

Immunotherapy is emerging as a promising tumor therapy, and NK‐cell‐mediated therapy offers a new treatment direction for leukemia and beyond leukemia. In the pre‐clinical and clinical stages, NK cell therapy has shown the ability to eradicate cancer cells and the potential for allogenic and off‐the‐shelf availability with minimal risk of toxicity or graft versus‐host disease (GvHD).^28^, ^29^ Taking these findings into account, recent studies have increasingly focused on optimizing the function of CAR‐loaded NK cells and extending the potential cell source, which paves the way for their clinical application.

Stable expression of therapeutic genes through genetic modification of NK cells, including CARs, is crucial for the development and downstream application of CAR‐NK immunotherapy. For example, transducing primary NK cells with a lentiviral vector is challenging, which also raises the safety concern of its non‐specific gene insertion. Nowadays, CRISPR/Cas9 is widely used for precise gene editing, whereas it is unable to neglect the high off‐target mutagenesis rate during clinical application.^30^, ^31^, ^32^ TALENickase, which produces single‐strand mutations by modifying TALEN, not only promotes homologous recombination efficiency but also displays lower cytotoxicity and off‐target mutations.^14^, ^33^ The human ribosomal gene region is specific, with high transcriptional activity and multiple copies, which is conducive to improving the efficiency of recombination.^15^ In this study, hiPSCs were engineered genetically and precisely to stably express an anti‐CD19 CAR using a non‐viral vector (minipHrneo), which was previously generated and confirmed for loading of the gene expression cassette at the rDNA locus by TALENickases‐mediated site‐specific integration.^16^, ^17^ The results demonstrated the high fidelity (no off‐target) and efficiency (35.7% and 30%) of gene editing by TALENickases at the rDNA locus of iPSCs.

In addition to the unique benefit of performing multiple accurate editing procedures at the single‐cell level, a customized functional iPSC production platform would facilitate the use of allogeneic “universal” cell therapy products that may be maintained in cell banks and offered when requested in a way similar to biopharmaceutical drugs to more broadly implement advanced cell therapy and reduce costs.^34^, ^35^ An increasing number of reports suggest that iPSCs can be obtained from various easily available sources, including skin and PB, and reprogrammed iPSCs can proliferate indefinitely in vitro without loss of pluripotency.^36^, ^37^, ^38^ Therefore, iPSCs can be used as an ideal resource for producing uniform NK cells of quality. Our research updated a published differentiation protocol to produce CAR‐iNK cells from the genetically engineered iPSCs.^39^ Due to the application of CEPT, the efficiency of EB generation and CD34^+^ production (72.4% vs. 53.9% and 63% vs. 51.4%, respectively) was elevated in the EB stage (Figures 2A and S3B), which was conducive for subsequent iNK cell generation, with the purity of CD3^−^CD56^+^NK cells as high as 95.3%.

To evaluate NK cells generated from iPSCs, we compared them with their native counterparts regarding functional capability and receptor expression. Similar to previous studies, it is revealed that iPSC‐derived NK and PB‐NK cells showed extremely identical characteristics besides the high expression of NKG2A in CAR19‐iNK cells,^19^, ^40^ which is usually expressed in more immature NK cells. Notably, CAR19‐IL24‐iNK cells express higher levels of CD57 and TRAIL, and the former is usually observed on highly mature cells in the CD56^dim^CD16^+^ NK cell population, which is defined as adoptive “memory” NK cells.^41^, ^42^ Besides, the latter involved in the TRAIL/TRAIL‐R pathway enables NK cells to mediate target cell death. These phenotypic traits suggested that acquiring more mature and functional NK cells with the improved protocol was successful.^12^, ^20^, ^43^

Various approaches have been established to expand NK cells in vitro. In the case of co‐culture with irradiated feeder cells, such as membrane‐bound IL21 and 4‐1BB ligands expressing K562 cells or irradiated human EBV‐LCL cells,^27^ the final CAR‐NK cell product carries a risk of contamination with co‐cultured cells. Therefore, the iNK cell expanding process should balance the product yield and purity against safety.^44^ In this regard, cytokine‐coated magnetic beads, which mimic feeder cells, seem safer and easier than removing feeder cells and are worthy of clinical applications despite the weaknesses of higher production.

Our results showed that CAR‐modified iNK cells displayed efficient killing power to CD19^+^ targets (Nalm‐6 cells) in relatively long‐term co‐culture and higher E:T ratio in vitro (Figure 5C,D), and there are encouraging advantages of co‐expressing IL24 to support the proliferation of CAR‐iNK cells both in vitro and in vivo (Figures S4E and 6D). IL24 sustains the survival and expansion of NK cells by autocrine stimulation, which may mediate growth and survival advantages, and further ameliorate the inherent limitation of the short duration of NK cells. IL24, belonging to the IL10 cytokine family, possesses a broad antitumor ability with no harm to normal cells.^45^, ^46^ To date, a majority of studies have focused on establishing IL24 for cancer therapy to kill tumor cells,^44^, ^47^, ^48^ while some studies reported that rIL24 is not capable of activating peripheral NK cells.^49^ In this study, it seems that rIL24 had no adverse effects on NK cells regarding cell survival and function. IL24 armored with CAR‐iNK cells showed enhanced proliferation and antitumor effect, and produced lower levels of IFN‐γ and GM‐CSF, which were correlated with a decreased risk of CRS and neurotoxicity compared to CAR19‐iNK cells, which might be safer following the prior reports and IL24 in lymphoma patients seems to be downregulated.^24^ The findings in this research suggest that the function of overexpressing IL24 may not be as uncomplicated as the exogenous addition of rIL24, especially in a complex in vivo environment. We presumed the potential risk of overexpressing IL24 in CAR‐IL24‐iNK cells is acceptable and likely with synergistic antitumor effects. Previous studies have elegantly shown that IL24 and IFN‐γ are regulated by the IFN‐STAT4 pathway in NK cells, while type I IFNs induce IFN‐γ expression upon NK cell activation, and then leads to the downregulation of IFN‐γ as STAT1 levels rise.^50^, ^51^ This may partially explain the elevated intracellular IFN‐γ level and lower secreted IFN‐γ level in CAR19‐IL24‐iNK cells in our experiments (Figures 5B and S4A,C). Moreover, the mechanism of IL24 in modulating the TME and immune response (such as the potential changes in the overall expression levels of IFN‐γ) is still under investigation.

In the further exploration of effector function transcriptional regulation in the terminal NK cell differentiation, it is possible to propose innovative strategies that can enhance differentiation and get more mature and functional iPSC‐derived NK cells. From the transcriptional standpoint, the genetically engineered iNK cells were remarkably similar. In comparison, pathways related to NK cells proliferation and antitumor activity, involving NFκB and Erk1/2, are upregulated significantly in CAR19‐IL24‐iNK cells, suggesting our success in the acquisition of functional NK cells. Moreover, our data revealed that CAR‐iNK cells brought about significant changes in the transcription of genes involved in multiple activating receptors and cytotoxicity effector molecules, including FASLG and TNFSF10 (TRAIL) (Figure 7A,D). Recent studies have confirmed that NK cells may improve immunity via inducing degranulation via TRAIL.^52^ Hence, it is reasonable that the changes we observed in CAR‐iNK cells might contribute to NK‐cell‐mediated cytotoxicity.

Despite this study providing a proof of concept that combining IL24 and CAR can effectively enhance the antitumor ability of iNK cells and suggest promising “off‐the‐shelf” cell products for immunotherapy, there are still some limitations in the approach. For instance, the results of our animal experiments did not satisfy our expectations. Different from the in vitro experiments, the complicated in vivo interactions make it challenging to elucidate through a single effect. However, it was a great challenge for CAR19‐IL24‐iNK cells function in our experimental strategy of “high tumor burden (10 × 10^5^) treated with low (3 × 10^6^) and dispersed (days 4, 11, and 18) therapeutic cell dose” compared to other reports.^53^ Thus, a consideration is to optimize the experimental design, adjust the therapeutic cell infusion dose, and advance the treatment window in subsequent studies. Additionally, recent studies have demonstrated that NK‐specific CAR (NK‐CAR) elements, including 2B4 and other NK cell activation receptors, could achieve optimal NK cell function, compared to T‐CAR (such as CD28‐41BBξ).^12^, ^54^ The introduction of NK‐CAR in our strategy would facilitate the potential for treating malignancies.

In summary, our results show that CAR19‐IL24‐iNK cells exhibit potent antitumor effects, which implies that overexpression of IL24 is a meaningful strategy for boosting CAR‐iNK cells’ antitumor effects and that iPSCs engineered with targeted integration of CAR19‐IL24 in the rDNA region may be accessible seed cells for generating next generation of iNK cells. At the same time, our exploration enriches the knowledge of the iPSC‐based CAR‐NK cells, features as off‐the‐shelf, and holds enhanced therapeutic potential, which expands their large‐scale clinical utilization to a wider range of patients against multiple malignancies.

## 4 MATERIALS AND METHODS

### 4.1 Cell culture

hiPSCs were cultured in mTeSR Plus medium (STEMCELL Technologies #100‐0276_C), which were previously reprogrammed from normal adult PB T cells by our laboratory. RelesR (STEMCELL Technologies #05872) was used to pass hiPSCs every 3‒4 days and plated onto plates covered with Matrigel (BD Biosciences #354277).

By the AMMS NK kit (T&L Biological Technology #AS‐22), the highly pure PB‐NK cells were acquired by activation and amplification in vitro. Four types of human tumor cell lines were used in this study, which includes Raji (Burkitt's lymphoma cell line that endogenously expresses CD19), Nalm‐6 (B‐cell acute lymphoblastic leukaemia [B‐ALL] cell line that endogenously expresses CD19), K562 (CML cell line, CD19^−^), and K562‐CD19 (CD19^+^, from Dr. Jie Liu) tumor cells. To monitor the tumor growth in the NSG xenograft model, Nalm‐6 (Luc1) was purchased from Meisen CTCC. All cell lines were cultured in Roswell Park Memorial Institute (RPMI) 1640 medium (Gibco #11875093) with 10% fetal bovine serum (FBS) (Sigma #F9423) and 1% Pen Strep (Gibco #15070063) (complete RPMI1640 medium) and regularly validated to be free of mycoplasma by PCR analysis. Cell culture was performed in a humidified environment with 5% CO_2_ at 37°C.

### 4.2 Construction of minipHrneo‐CAR19/CAR19‐IL24 plasmid and generation of CAR‐modified hiPSCs

Previous studies have constructed minipHrneo, a non‐viral hrDNA targeting vector, which involved non‐promoter neomycin (Neo) cassette (for screening) and a synthesized *CAR19‐IL24* fragment.^17^, ^55^ In this study, the *CAR19‐IL24* expression cassette was introduced into the appropriate restriction site of minipHrneo to constitute minipHrneo‐*CAR19‐IL24*. Sanger sequencing was used to verify the final product. All restriction enzymes were purchased from New England Biolabs (Beverly). All PCR and ligation reaction kits were purchased from Takara Bio.

For gene targeting, hiPSCs were dissociated into single‐cell with TrypLE Select (Invitrogen #12563029) at 37°C for 3 min and counted. A total of 3 × 10^6^ cells were collected in 100 µL Human Stem Cell Nucleofector Kit 2 solution (Lonza #VVPH‐5022) containing 5 µg of minipHrneo‐*CAR19/CAR19‐IL24* and 5 µg of TALEN/TALENickase.^14^, ^17^ The program B‐016 on Nucleofector II (Lonza #AAB‐1001) was used for completing nucleofection. The transfected cells were cultured with mTeSR Plus medium supplemented with 10% Clone R (STEMCELL Technologies #05888). Three days post‐transfection, integrated hiPSCs were selected with mTeSR Plus medium adding 50 µg/mL G418 (Sigma #A1720) for 7 days. Then, G418‐resistant clones were isolated for subsequent identification and application.

LV‐CAR19‐iPSCs were transduced as a control in the subsequent assays. CAR19 expressing lentivirus was constructed and the lentiviral particles were produced as noted.^55^ With a multiplicity of infection of 20, the produced hiPSCs in 10 cm dishes were transduced with the optimum quantity of concentrated lentiviral supernatants supplemented with 0.8 µg/mL of polybrene for 6 h. The transduced iPSCs were cultured in fresh mTeSR Plus medium overnight and collected for the following studies.

### 4.3 Identification of site‐specific integration CAR‐iPSC colonies

Colonies of G418‐resistant hiPSCs were identified by Southern blotting, Sanger sequencing, and PCR. Details about it are shown in the Supporting Information.

### 4.4 Karyotype analysis of iPSCs

For karyotype analysis, a G‐banding analysis of chromosomes from each iPSC line was performed. The iPSCs were trypsinized and pelleted after treatment with 0.1 µg/mL colcemid (Sigma) for 4 h and then treated with 0.075 M KCl at 37°C for 10 min. The air‐drying method was used to prepare metaphase chromosome spreads after fixation with Carnoy fixative. The G‐banded analyzed chromosomes were stained with Giemsa (Sigma #48900) and analyzed under the microscope.

### 4.5 Characterization of iPSCs

Immunofluorescence staining of iPSC surface markers was performed.^56^ OCT4 (Abcam #ab181557), NANOG (Abcam #ab109250), SOX2 (Proteintech #11064‐1‐AP), and SSEA‐4 (Merck Millipore #SCR001) were used to incubate cells with 1:100 diluted primary antibodies in 5% bovine serum albumin (BSA) at 4°C overnight. 4′,6′‐Diamidino‐2‐phenylindole (Bioss) was selected for nuclear staining. Fluorescence microscope was used to capture images of stained cells. (Leica DM IRB).

### 4.6 Generation and characterization of iPSC‐derived NK cells

Briefly, a two‐step protocol was adopted via EB formation. According to the instructions, iPSCs were seeded onto the Aggrewell 800 (STEMCELL Technologies #34850). After a 10‐day half‐medium change with EB formation medium, EBs were transferred to gelatin‐coated dishes, and half‐medium changes were made twice a week after the first week. To remove any clumps, cells can be collected via a 40 µm cell strainer (Corning #431750).

Biotinylated IL21 (Acro Biosystems #IL1‐H82F7) and biotinylated 4‐1BBL (Acro Biosystems #41L‐H82F9) bound streptavidin magnetic beads (Invitrogen #91216845) were employed to stimulate the expansion of NK cells. Beads were added to NK cells at a 1:1 ratio. Medium was replaced every 2‒3 days with NK cell expansion medium freshly added with 500 IU/mL IL2 and 10 ng/mL IL15. The coupling results of beads were tested using flow cytometry: IL21 (eFlour660; eBioscience) and 4‐1BBL (PE‐CY7; eBioscience).

To characterize the iPSC‐derived NK cells, flow cytometry was used to evaluate the expression level of the NK cell surface markers. The cell culture medium and antibodies are listed in the Supporting Information.

### 4.7 Quantitative real‐time PCR

RNA was extracted with TRIzol reagent (Invitrogen #15596018), and RNA quality and quantity were measured with NanoDrop 2000 (Thermo Fisher Scientific). RNA was converted into cDNA by RT Mix (Vazyme) and processed for quantitative real‐time PCR based on the CFX96 system (Bio‐Rad #1855201) by SYBR Mix (Vazyme). The results were normalized to the level of 18s, and the ΔΔCT method was used to calculate relative expression levels. The following primers were used for real‐time PCR experiments:

FMC63‐F: 5′‐GTGGCTGGGAGTAATATGGG‐3′
FMC63‐R: 5′‐GGAGACGGTGACTGAGGTTC‐3′
IL24‐F: 5′‐CTTCTGGGCTGTGAAAGACACTATGC‐3′
IL24‐R: 5′‐CTGGGTTGCAGTTGTGACACGATG‐3′
Ribosomal 18s‐F: 5′‐F AACCCGTTGAACCCCATT‐3′
Ribosomal 18s‐R: 5′‐CCATCCAATCGGTAGTAGCG‐3′

### 4.8 Co‐culture cytotoxicity assay

The cytotoxicity of CAR‐iNK cells was detected by co‐culture with tumor target cells (Nalm‐6, Raji, K562, and CD19‐K562) via enzyme release assay, fluorescence‐labeled flow assay, and IncuCyte real‐time image system (Essenbioscience). Details about the above experiments are listed in the Supporting Information.

### 4.9 Western blotting

The cells were washed with ice‐cold phosphate‐buffered saline twice, followed by lysis using RIPA buffer (Bioss) supplemented with the protease inhibitor phenylmethylsulfonyl fluoride (ApexBio) and the protease inhibitor cocktail (ApexBio). By the bicinchoninic acid (BCA) protein assay kit (Thermo Fisher), the protein concentrations were decided. The concentrated proteins were mixed with loading buffer and denatured by heating. Equal amounts of proteins were separated through the sodium dodecyl sulfate polyacrylamide gel (SDS–PAGE) and transferred onto the polyvinylidene fluoride membrane (Merck Millipore). Membranes were then blocked with 5% (w/v) bovine albumin (BSA) and/or non‐fat milk in tris buffered saline containing 0.15% Tween 20 (TBST). Incubated the membranes with specific primary antibodies at 4°C overnight and a suitable secondary antibody for 1 h successively. Protein bands were visualized by employing chemiluminescence (Thermo Fisher Scientific). Band intensity was analyzed via image analysis software Image Lab (Bio‐Rad Laboratories). By staining GAPDH, each loading sample was normalized. The Supporting Information presents the antibodies adopted for Western blotting.

### 4.10 ELISA assays

Cytokine levels produced by four iPSC‐derived NK cells were detected using a human IL24 (Abclonal, #RK00112), GM‐CSF (Abclonal, # RK04505), TNF‐α, and IFN‐γ ELISA kit (Invitrogen #KHC4021, #BMS2034) according to manufacturer instructions. A total of 1 × 10^6^ iNK cells were seeded with the stimulus of PMA (TopScience; #TQ0198) and ionomycin (TopScience; #T7285). Abiding by the incubation of 37°C, 24 h, cell‐free culture supernatants were used for detection. ELISA was performed with two technical replicates for all samples. Measurements were performed in triplicates.

### 4.11 NSG xenograft model

M‐NSG mice (No. NM‐NSG‐001) were purchased from Shanghai Southern Model Biological Research Center. The mice were allocated into six groups (*n* = 4) at random and earmarked after 1 week of adaptive feeding. On day 0, male NSG mice (6‒8 weeks old, about 25 g) were inoculated intravenously (i.v.) with 1 × 10^6^ Nalm‐6 (Luc1) cells (Meisen CTCC). On each of days 4, 11, and 18, mice were treated with tail‐vein injection (3 × 10^6^) of the iNK cells. Tumor growth was assessed by monitoring tumor bioluminescence and body weight at regular intervals. BLI was performed using PerkinElmer In Vivo Imaging System (Perkin Elmer) on days 7, 10, 14, 19, 24, and 28 separately. The body weight was recorded every 3 days after injection and compared relative to the initial body weight. On day 28, mice were euthanized by CO_2_ inhalation. All the mice were housed in standard cages, with six mice per cage under relatively stable conditions (12‐h light and 12‐h darkness, 70% humidity, 22°C‒24°C). Diets and ultrapure water were provided.

### 4.12 RNA‐sequencing and analysis

The iNK cells were obtained and submitted to Shanghai Xuran Biological Company for RNA isolation, sequencing, and analysis. Raw data can be accessed in the GEO database (GSE260573). For better comparison, further raw data files of fresh PB‐NK cells and activated PB‐NK cells (GSE165498) were downloaded from the GEO database. Gene expression levels were estimated based on read counts and fragments per kb per million reads values by employing HTSeq (version 0.6.1) and then normalized (log2). Differential expression analysis of different NK cell groups was performed using the DESeq2 R package (version 2_1.6.3). Functional analysis of DEGs was performed using Gene Ontology (GO) and KEGG databases. Following the MSigDB and the GO database, the GSEA and over‐representation analysis were performed.

### 4.13 Graphs and statistical analysis

All statistical analyses in this study were performed using Prism8 (GraphPad Software). As the figure legends stated, for comparing the two experimental groups with normally distributed data, an unpaired two‐tailed Student's *t*‐test was employed. Two‐way analysis of variance (ANOVA) followed by Dunett's multiple comparison test or one‐way ANOVA with Bonferroni correction for pairwise comparison for normal distributions was used to compare the various experimental groups. Non‐normally distributed data was explored via the Mann‒Whitney test. All data are presented as the mean ± standard deviation (SD). Survival data were compared by using the log‐rank test and analyzed via the Kaplan‒Meier method. Via the Holm‒Sidak method, *p*‐values were decided by the two‐way ANOVA with repeated measures and adjusted. Data were presented as mean ± SD and *p* < 0.05 was considered statistically significant. *p*‐Values were indicated with asterisks: ns, not significant, *p* > 0.05; ^*^ *p* < 0.05, ^**^ *p* < 0.01, ^***^ *p* < 0.001, ^****^ *p* < 0.0001.

## AUTHOR CONTRIBUTIONS

D.L., L.W., and Q.H. were responsible for the design and oversight of the study as well as the editing of the manuscript. Y.Z. conducted most of the experiments and wrote the manuscript. Q.S. performed partial experiments and analysis and interpretation of data. P.W., C.H., S.T., and M.Z. contributed to the preparation of the manuscript. All the authors reviewed and approved the final version of the manuscript.

## CONFLICT OF INTEREST STATEMENT

The authors declare they have no conflicts of interest.

## ETHICS STATEMENT

The study was performed following the Declaration of Helsinki. The animal study was reviewed and approved by the Department of Laboratory Animals, Central South University (CSU‐2022‐0247 and CSU‐2022‐0001‐0260), and peripheral blood was collected from the volunteers with an informed consent form and approved by the Ethics Committee, School of Life Sciences, Central South University (No. 2021‐1‐21).

## Supporting information

Supporting Information

## Data Availability

Supporting data are provided by the corresponding author upon reasonable request.
