# Supplementary material for: iPSC‐derived NK cells with site‐specific integration of CAR19 and IL24 at the multi‐copy rDNA locus enhanced antitumor activity and proliferation
Source: MedComm (2020). 2024 May 9;5(5):e553. doi: 10.1002/mco2.553 (PMC11082533; doi:10.1002/mco2.553)
Supplement: Supplementary file 1 — Supporting Information [file MCO2-5-e553-s001.docx]

**iPSCs-derived NK cells with site-specific integration of CAR19 and IL24 at the multi-copy rDNA locus enhanced antitumor activity and proliferation**

**Yuxuan Zhang^1,#^, Qingxin Shi^1,#^, Peiyun Wang^1^, Chujun Huang^1^, Shuqing Tang^1^, Miaojin Zhou^1^, Qian Hu^1,*^, Lingqian Wu^1,*^ and Desheng Liang^1, 2*^**

1 Center for Medical Genetics & Hunan Key Laboratory of Medical Genetics, School of Life Sciences, Central South University, Changsha, Hunan, 410078, China

2 Hunan Key Laboratory of Animal Models for Human Diseases, School of Life Sciences, Central South University, Changsha, Hunan, 410078, China

# These authors contributed equally to this work.

* Corresponding author(s):

Qian Hu: [huqian@sklmg.edu.cn](mailto:huqian@sklmg.edu.cn)

Lingqian Wu: [wulingqian@sklmg.edu.cn](mailto:wulingqian@sklmg.edu.cn)

Desheng Liang: [liangdesheng@sklmg.edu.cn](mailto:liangdesheng@sklmg.edu.cn)

**This file includes:**

Supplementary Figure 1 to 5

Detailed Methods

Running title: Exploration of IL24 armored CAR-iNK cells


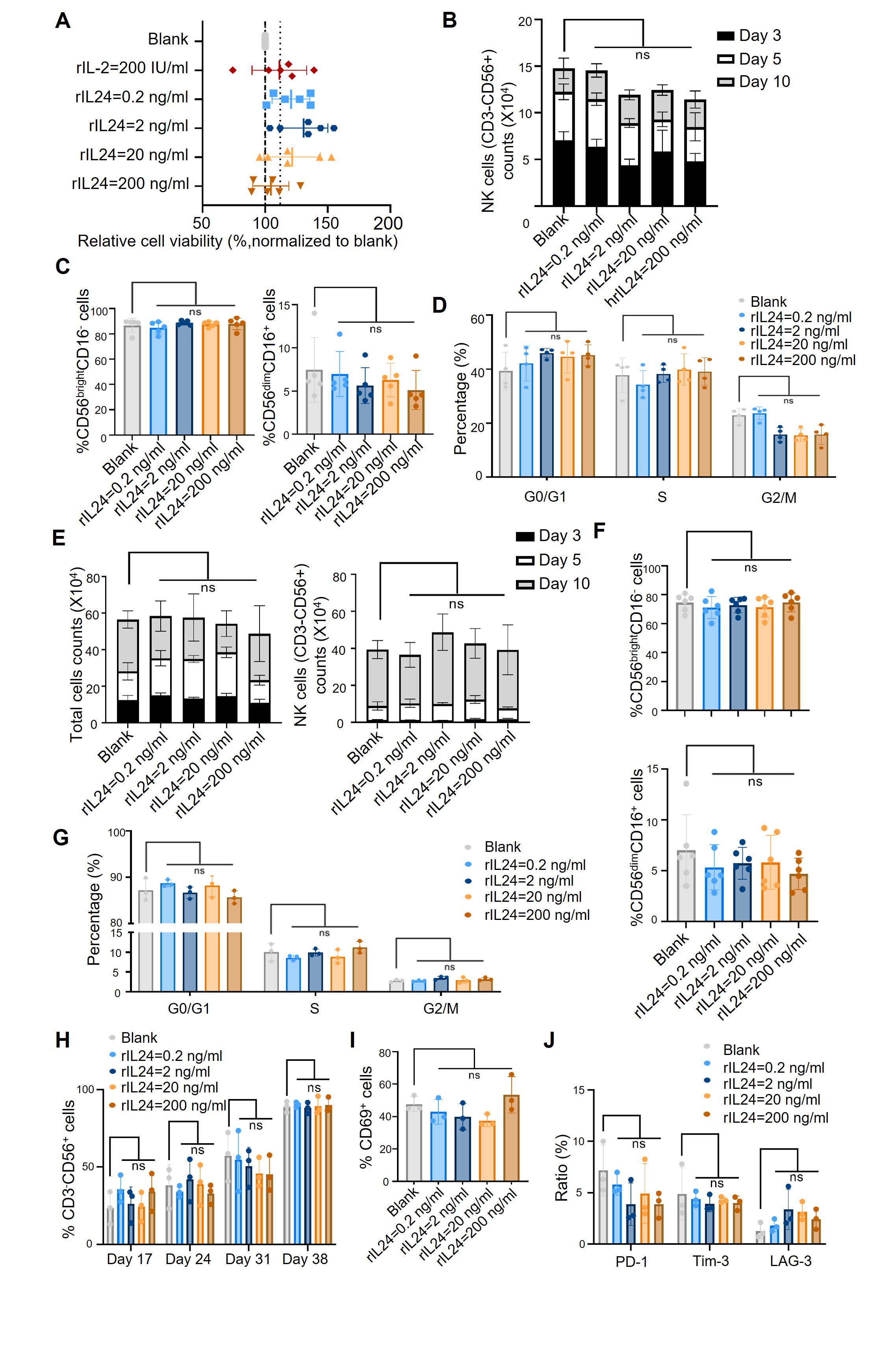


**Supplementary Figure 1. No adverse effects on proliferation, subpopulation composition and cell cycle of NK cells and differentiation of iNK cells treated with rIL24.** (A) Isolated and purified human peripheral blood NK cells (CD56^+^%>95%) were treated with different concentrations of human rIL24 (0, 0.2, 2, 20, 200 ng/ml) in the absence of human rIL-2, and cell viability was measured by CCK8 after 2 days. rIL-2 treatment group (200IU/ml) was used as a control. Data are representative of at least six independent experiments. (B~D) Effects of rIL24 on proliferation, subpopulation composition and cell cycle of CD3-CD56+ NK cells. (B) Purified NK cells were treated with different concentrations of rIL24 in the absence of rIL-2 and counted on day 3, 5 and 10 using Countstar. On day 10, the distribution of NK cell subpopulations (CD56^bright^CD16^-^ and CD56^dim^CD16^+^) (C) was detected by flow cytometry, and the cell cycle (D) was detected by PI staining assays. Data are representative of five independent experiments in (B~C) and four independent experiments in (D). (E~G) Effects of rIL24 on proliferation, subpopulation composition and cell cycle of PBMCs. Fresh PBMCs were treated with different concentrations of rIL24 (0, 0.2, 2, 20, 200 ng/ml) in the absence of rIL-2, total cells were counted by Countstar and the proportion of NK cells (CD3^-^CD56^+^) in the culture system was detected by flow cytometry (E). On day 10, the distribution of NK cell subpopulations (F) was detected by flow cytometry, and the cell cycle (G) was detected by PI staining assays. Data are representative of at least six independent experiments in (E~F) and three independent experiments in (G). (H~J) Effects of rIL24 on the differentiation process of iPSCs into NK cells. The differentiation process of iPSCs into NK cells was treated with different concentrations of rIL24, and the proportion of iNK (CD3^-^CD56^+^) cells in suspended cells was evaluated by flow cytometry on day 17, 24, 31 and 38, respectively (H), and the expression level of CD69 (I) and immune checkpoint (J) on the cell surface was detected by flow cytometry on day 38. Data are representative of at least three independent experiments.

GraphPad Prism8 was used to analyze all data with error bars, which were expressed as mean ± standard deviation (SD). Two-way ANOVA with Dunett correction, ns, not significant.


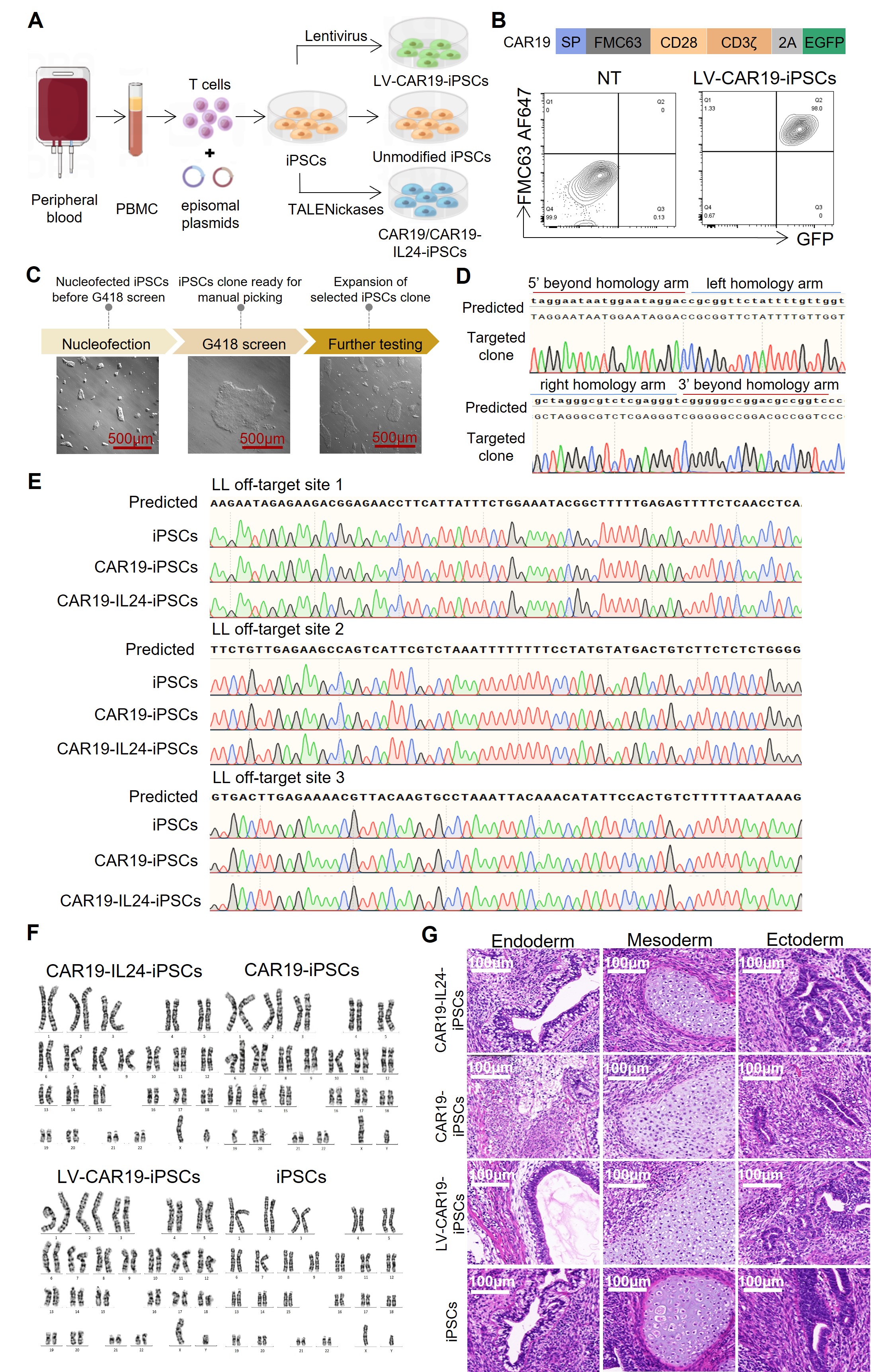


**Supplementary Figure 2. Generation and characterization of CAR-loaded iPSCs.** (A) Schematic diagram of iPSCs reprogramming from the peripheral blood T cells by using an episomal reprogramming strategy. PBMC, Peripheral blood mononuclear cell. (B) Schematic diagram of FMC63scFv-28ζ (CAR19) and representative flow cytometry data showed co-expression of CAR19 and EGFP in LV-CAR19-iPSCs post-transduction. SP, signal peptide. 2A, 2A “self-cleaving” peptide. EGFP, enhanced green fluorescent protein. NT, negative. (C) Morphology of representative iPSCs after nucleofection, manual selection, and culture of monoclones (scale bar = 500 µm). (D) Sanger sequencing of homologous recombination. It was used to further validate two PCR products, which were consistent with the expected theoretical sequence. (E) Analysis of potential off-target sites. Sanger sequencing for CAR19-IL24-iPSCs, CAR19-iPSCs, LV-CAR19-iPSCs and iPSCs to examine the prediction of off-target sites in gene-edited clones. (F) Karyotypes of CAR19-IL24-iPSCs, CAR19-iPSCs, LV-CAR19-iPSCs and iPSCs. The transformed iPSCs clones showed a normal karyotype (46, XY) consistent with the normal iPSCs. n=5 for each group. (G) Tissue sections of iPSCs teratoma were stained with HE. Tissue originating from three germ layers (ectoderm, mesoderm and endoderm) was found in the tumors formed in the NSG mice injected with four types of iPSCs. (scale bar=100 µm)


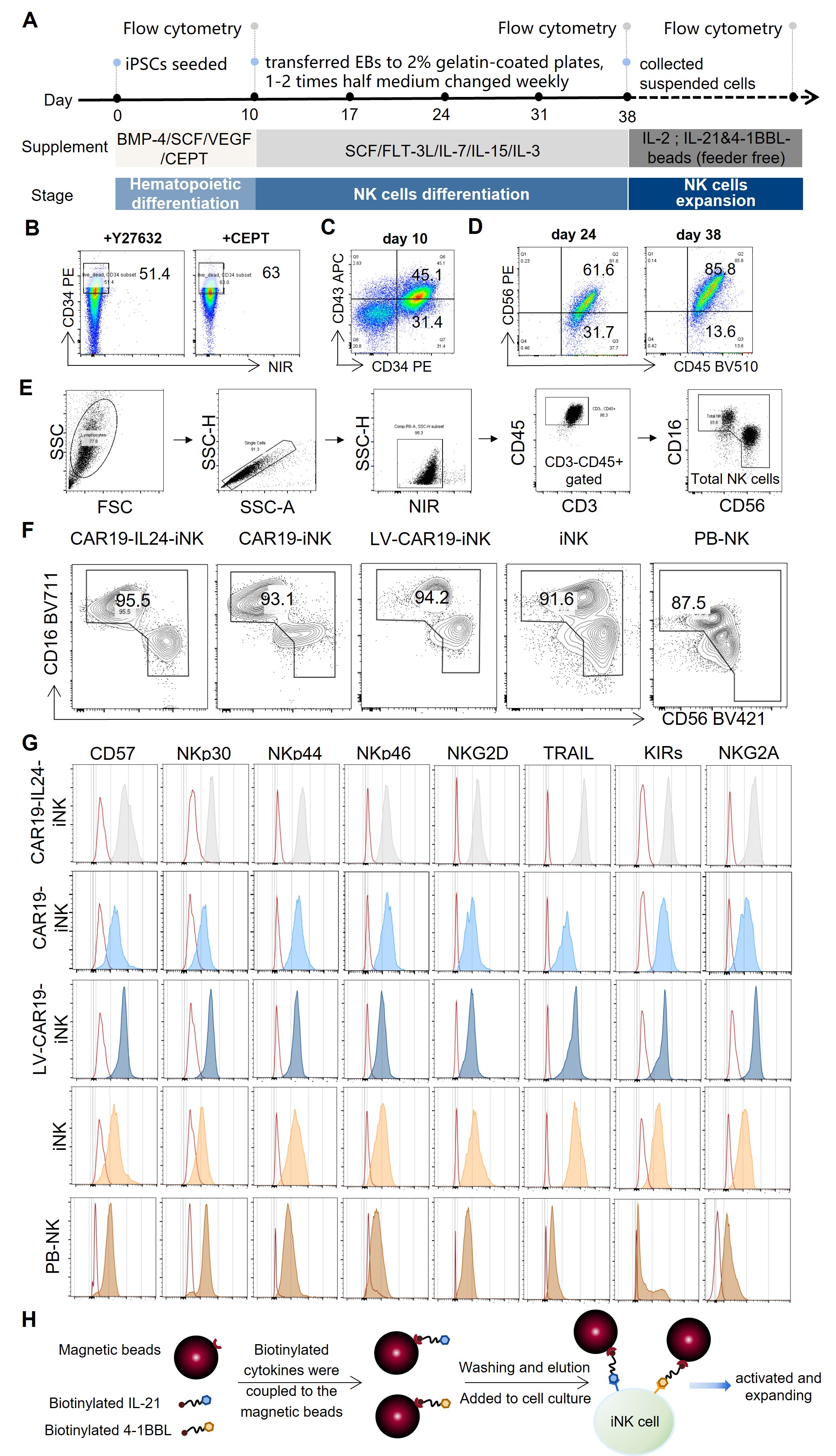


**Supplementary Figure 3.** **Characterization of iPSC-derived iNK cells.** (A) Flow chart of iPSC-derived NK cells differentiation and expansion procedures. iPSCs were seeded onto the Aggrewell™800 on day 0. After a 10-day half-medium change with EB formation medium, EBs (embryoid bodies) were transferred to gelatin-coated dishes, and half-medium changes were made twice a week after the first week. Biotinylated IL-21 and biotinylated 4-1BBL bound Streptavidin magnetic beads were used to stimulate the expansion of NK cells since day 38. NK cell surface markers were detected by flow cytometry on day 10, day 38, and during the expansion period. (B) Comparison of EB formation and yield using small molecules (CEPT vs. Y-27632) in epithelial cells-derived iPSCs. (C~D) Analysis of the proportion of CD34/CD43 cells of EBs (day 10) (C) and proportion of CD45^+^CD56^+^ cells during differentiation (day 24 and day 38) (D) by flow cytometry. (E) Gating strategy of FCM assays used in suspension cells at day 38. (F) Representative flow cytometry data showed the total NK cells (CD56/16 composition) of four differentiated iNK cells with CD45^+^CD3^-^ gated. (G) Representative flow cytometry showed surface expression of NK cell markers. PB-NK as the control. The red line refers to the isotype control. Data represent 3 independent experiments. (H) Schema diagram showing the preparation of biotinylated protein-coupled by streptavidin magnetic beads and iPSC-derived NK cells expansion in vitro. Biotinylated IL-21 and biotinylated 4-1BBL were coupled to the Streptavidin magnetic beads to stimulate the expansion of NK cells. Beads were added to NK cells at a ratio of 1:1.


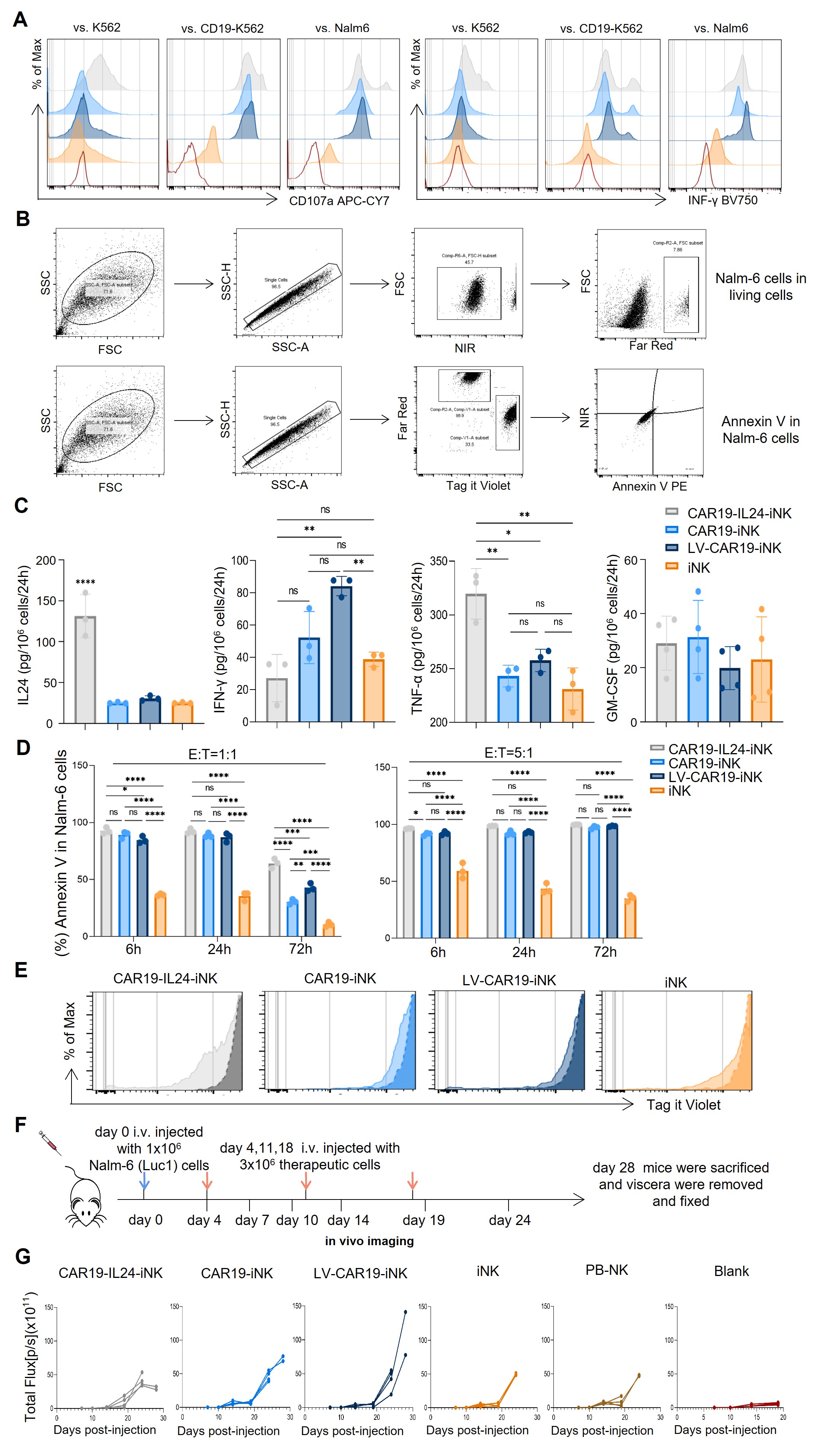


**Supplementary Figure 4.** **CAR19-IL24-iNK cells exhibited a more effective killing effect.** (A) Release of CD107a and IFN-γ after being challenged with K562 (CD19^-^), CD19-K562 (CD19^+^) and Nalm-6 (CD19^+^) cells. Representative flow cytometry data were shown. (B) Gating strategy of flow cytometry assay used in NK cells killing assay. (C) Histogram analysis of cytokine production in the culture supernatant. Cell-free supernatants of iPSC-derived NK cells were harvested after 24h incubation with the stimulus of PMA and ionomycin. Human IL24, TNF-α, IFN-γ and GM-CSF were measured by ELISA. Data represent 3 independent experiments for IL24, IFN-γ, TNF-α, and 4 independent experiments for GM-CSF. (D) Quantitative and statistical results showed the apoptosis in Far Red-labeled Nalm-6 cells. (E) Representative flow cytometry of dilution in iPSC-derived NK cells labeled with Tag-it Violet after 72 h with an effector: target ratio of 5:1. The dashed line indicated the labeled iNK cells at 0 h, while the light line indicated the labeled iNK cells at 72 h. (F) Schema of the NSG xenograft model. On day 0, NSG mice were inoculated intravenously (i.v.) with Nalm-6(Luc1) cells (1×10^6^). On day 4, 11 and 18, mice were treated with tail-vein injection (3×10^6^) of DiR-labeled iPSC-derived NK cells. On day 28, mice were euthanized by CO_2_ inhalation. (G) Quantification of tumor burden over time for each animal over time (n = 4), represented by total flux (photons/s). Tumor growth in the xenograft model was monitored by measuring changes in tumor bioluminescence on Nalm-6(Luc1) day 7, 10, 14, 19, 24 and 28.

GraphPad Prism8 was used to analyze all data with error bars, which were expressed as mean ± standard deviation (SD). (C) One-way ANOVA with Bonferroni correction, ns, not significant; p > 0.05; * p < 0.05, ** p < 0.01, *** p < 0.001, **** p < 0.001.


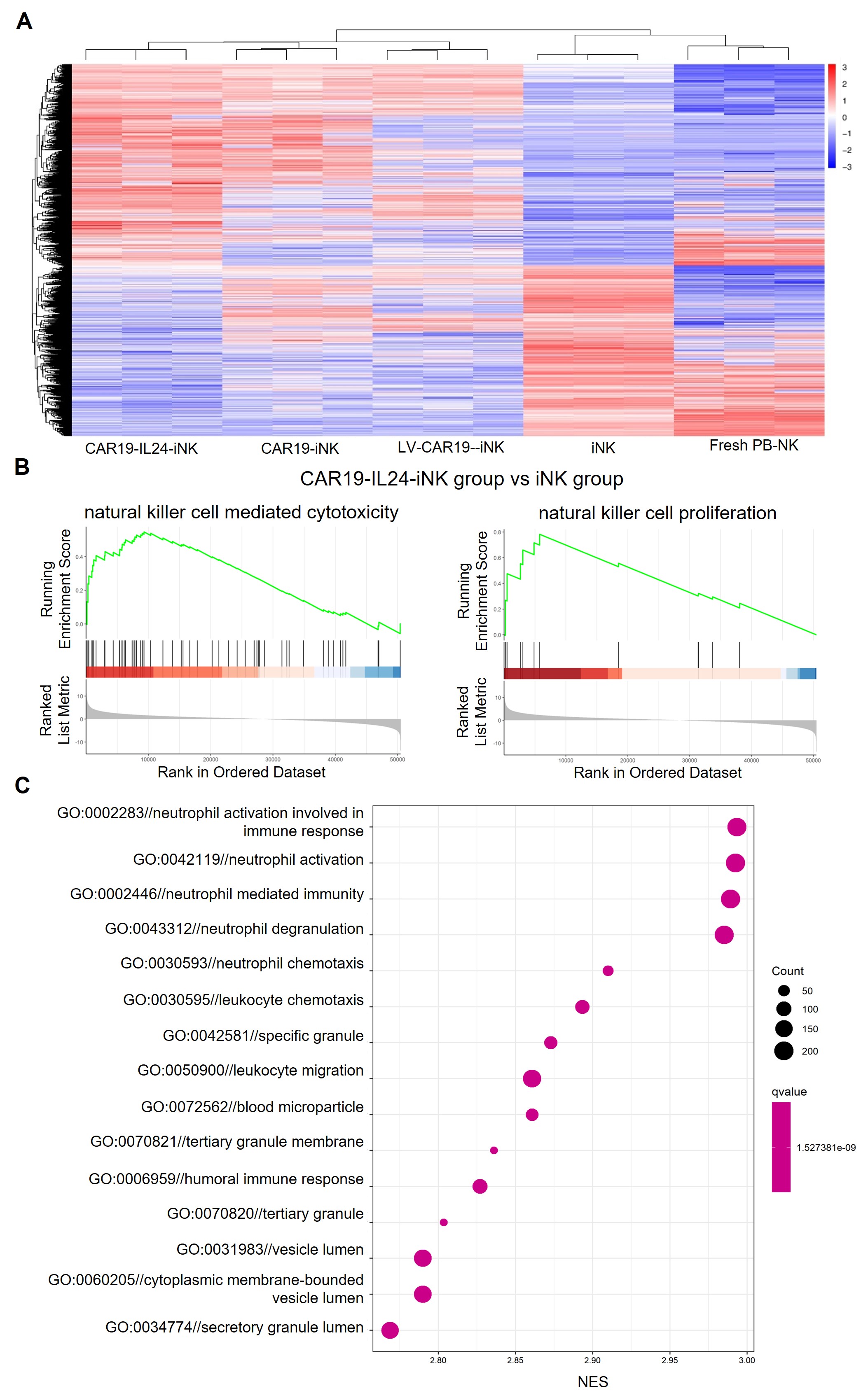


**Supplementary Figure 5. IL24 armored CAR-iNK cells promote NK cell-mediated immunity via activating NFκB and Erk1/2.** (A) Unsupervised gene clustering of various NK cell populations. n=3 for each group. (B) GO terms of biological processes enriched in CAR19-IL24-iNK cells versus iNK cells by GSEA analysis. n=3 for each group. (C) Bubble plot showing gene set enrichment analysis (GSEA) and over-representation analysis (ORA) of gene sets related to NK cells proliferation and functions.

**Detailed Methods**

***1. Identification of site-specific integration CAR-iPSCs colonies***

Colonies of G418-resistant hiPSCs were identified by Southern blotting, Sanger sequencing, and PCR. Details about it were noted in the Supplementary files.

The standard phenol/chloroform extraction method was used to isolate genomic DNA from iPSCs. PCR was conducted using LA Taq DNA polymerase (TaKaRa #RR002A). The site-specific integration colonies were identified using primers, Up-screen-F/Up-screen-R and Down-screen-F/Down-screen-R, with an annealing temperature of 58°C and 56°C, respectively. Sanger sequencing was used to identify all the PCR products. The primer sequences are as follows:

Up-screen-F: 5’-CCTGAGAAACGGCTACCACATCC-3’

Up-screen-R: 5’-TTTCCACAACTATCCAACTCACAACG-3’

Down-screen-F: 5’-GAGCATTCAAACAGTTGGACG-3’

Down-screen-R: 5’-GATCCACCGCTAAGAGTCGTAC-3’

Non-directed integration clones were excluded by Southern blotting: 10 µg of genomic DNA was digested overnight with XhoI and KpnI restriction enzymes (New England Biolabs #R0146, #R3142L), followed by electrophoresis on a 0.8% agarose gel at 120 V for 3 hours. Capillary transferring of 0.4 M NaOH was used to transfer the digested DNA fragment onto a positively charged nylon membrane (Roche Diagnostics #11209299001, Basel, Switzerland). DIG-labeled λ-HindIII DNA Marker (Roche Diagnostics) was adopted as the molecular weight marker. DIG-dUTP-labeled probes were used to hybridize blots on the nylon membrane at 42°C overnight. CDP-Star (Roche Diagnostics) was used as a chemiluminescent substrate to measure signals after incubation with AP-conjugated DIG antibody (Roche Diagnostics) and washing. Probes were generated using a PCR DIG Probe Synthesis Kit (Roche Diagnostics) with probe-F/R primers. The length of probes was 358 bp and the sequences are as follows.

probe-F: 5’-CCGGTCTTGTCGATCAGGATGA-3’

probe-R: 5’ -CAGAGTCCCGCTCAGAAGAACT-3’

***2. Cell culture medium for iPSC-derived NK cells***

**EB formation medium:** STEMdiff™ APEL™2 Medium (STEMCELL Technologies #05270) supplied with 40 ng/mL SCF (Sino Biological #10451-H08B), 20 ng/mL BMP4 (Peprotch #120-05ET) and 20 ng/ml VEGF (Sino Biological #29656-HNAB). 70 μM Trans-isrib (Cayman #16258), 5 μM Emricasan (MedChemExpress #10396), and 50 nM Chroman 1 (MedChemExpress #HY-15392) were added on the first day.

**NK cell differentiation medium:** 55.3% DMEM + GlutaMAX-I (Gibco #10566016), 27.7% F12 + GlutaMAX-I (Gibco #31765035), 15% fetal bovine serum (Sigma #F9423), 1% P/S (Gibco #15070063), 2 mM L-glutamine (Gibco #21051024), 25 uM β-mercaptoethanol (Sigma #M6250), 5 ng/mL sodium selenite (Sigma #S5261), 50 uM ethanolamine (MP Biomedicals #194658), 20 mg/L ascorbic acid (Sigma #A5960), 5 ng/mL IL-3 (Sino biological #11858-HNAE), 20 ng/mL SCF (Novo Protein, Catalog #C034), 20 ng/mL IL-7 (Novo Protein #CX47), 10 ng/mL IL-15 (Acro Biosystems #IL5-H4117), and 10 ng/mL Flt3 ligand (FLT3L) (Acro Biosystems #FLL-H5218).

**NK cell expansion medium:** NK MACS medium (Miltenyi #130-114-429), 2.5% supergrow (DAKEWE #6122012), 1% P/S and 500 U/mL IL-2 (T&L Biological #GMP-TL777).

***3. Effect of IL24 on NK cells***

Isolated and purified human peripheral blood NK cells (CD56^+^%>95%) and fresh PBMCs were treated with different concentrations of human rIL24 (0, 0.2, 2, 20, 200 ng/ml) in the absence of human rIL-2 and counted using Countstar. Different concentrations of rIL24 were added during the differentiation of iPSCs into NK cells. NK or iNK cells were stained for cell-surface markers to determine phenotypes with the following antibodies: CD3 (AF700; BioLegend), CD45 (BV510; BioLegend) CD56 (PE; BioLegend), CD16 (BV711; BioLegend), CD69 (PerCP eFluor 710; eBioscience), PD-1 (PE/Fire700; Biolegend), Tim-3 (PE; Biolegend), LAG-3 (PerCP/Cy5.5; Biolegend).

Cell viability was measured by CCK8 (Beyotime, C0037) after 2 days. To analyze cell cycle, 1×10^6^ cells were collected, washed once and fixed in cold 70% ethanol at 4°C overnight. Then, fixed cells were stained with Propidium iodide (PI) (Biolegend, 421301) according to the manufacturer’s instructions.

***4. Characterization of iPSCs-derived NK cells***

The expression of the following NK cell surface markers was typically tested using flow cytometry: CD3 (AF700; BioLegend), CD45 (BV510; BioLegend) CD56 (BV421; BioLegend), CD57 (FITC; BioLegend), CD16 (BV711; BioLegend), NKP30 (BV605; BioLegend), NKP44 (PerCP-Cy5.5; BioLegend), NKP46 (BV786; BD), KIRs (PE; BioLegend), NKG2D (PE-Cy7; BioLegend), TRAIL (APC; BioLegend), NKG2A (PE-Cy5; BioLegend), FMC63 (PE; ACRO), and FMC63 (AF647; ACRO).

***5. Co-culture cytotoxicity assay***

The cytotoxicity of NK cells was detected via enzyme release assay and fluorescence-labeled flow assay. The iPSC-derived NK cells were labeled with Tag-it Violet™ dye (BioLegend #425101) and tumor target cells (Nalm-6, Raji, K562, CD19-K562) with CellTrace™ Far Red (Invitrogen #C34572). They were then used in the following experiments.

**Lactate dehydrogenase release-based cytotoxicity assay**

To detect the cytotoxicity of iPSC-derived NK cells against Raji, Nalm-6, K562-CD19, and K562 cells, target cells (0.8, 0.6, and 0.2 × 10^4^) were seeded in each well of a 96-well V-bottom plate (Corning #3357). The iNK cells were added at different effector-target ratios (0.5, 1, and 5) and then co-cultured in complete RPMI1640 medium without cytokines. After 18 hours of incubation, the cell-free supernatant was gathered through centrifugation. The release of lactate dehydrogenase (LDH) in the supernatant was tested by Lactate dehydrogenase assay kit (Nanjing Jiancheng #A020-2-2, Nanjing, China).

**Cytotoxic factor release assay**

The cells obtained in the previous part stimulated in vitro supplemented with monensin were treated with fixation and permeabilization, stained, and tested for cytotoxic factor levels: CD107a (APC-CY7; BioLegend), IFN-γ (BV750; BioLegend), by flow cytometry using BD Cytofix/Cytoperm™ Fixation/Permeabilization Kit (BD #554715).

**Flow cytometry-based cytotoxicity assay (FCbCAssay)**

To detect the interaction and toxicity between iPSC-derived NK cells and tumor cells during co-culture, a flow cytometry-based cytotoxicity assay (FCbCAssay) was designed. Briefly, iNK cells and tumor target cells were mixed at the ratio of 1:1/5:1 and co-cultured in complete RPMI1640 medium without rIL-2. Aliquots of well-mixed cultures were seeded in a 96-well plate and tested by flow cytometry after 6 h, 24 h and 72 h. Specifically, cells were harvested and stained by Annexin V-PE/7-AAD Apoptosis Detection Kit (Vazyme #A213-01). Next, the respective apoptosis levels were analyzed of tumor cells or iNK cells labeled respectively, and the staining intensity of iNK cells was also compared to determine the proliferation level by flow cytometry. Data analysis was performed using FlowJo (Treestar, Ashland, OR).

**IncuCyte-based cytotoxicity assay**

Nalm-6 cells(labeled with Incucyte® Cytolight Rapid Green Dye) cocultured with effect cells (labeled with Incucyte® Cytolight Rapid Red) with variant E:T ratio monitored by Incucyte real-time image system (Sartorius) over 72h. Apoptosis and proliferation were measured by counting the pre-stained nuclei.

***6. Antibodies for immunoblots***

Anti-hIL24 (proteintech, #12064-1-AP), Anti-CD3ζ (GeneTex, #GTX108479), Anti-NFκB (proteintech, #10745-1-AP), Anti-phospho-NFκB (Huabio, #ET1604-27), Anti-Erk1/2 (proteintech, #11257-1-AP), Anti-phospho-Erk1/2 (Huabio, #ET1610-13), Anti-GAPDH (Sigma, #G9545)
